# Medical Management and Device-Based Therapies in Chronic Heart Failure

**DOI:** 10.1016/j.jscai.2023.101206

**Published:** 2023-12-04

**Authors:** Andrew H. Nguyen, Madelyn Hurwitz, Jacob Abraham, Vanessa Blumer, M. Casey Flanagan, A. Reshad Garan, Manreet Kanwar, Rachna Kataria, Jamie L.W. Kennedy, Ajar Kochar, Jaime Hernandez-Montfort, Mohit Pahuja, Palak Shah, Matthew W. Sherwood, Behnam N. Tehrani, Saraschandra Vallabhajosyula, Navin K. Kapur, Shashank S. Sinha

**Affiliations:** a Inova Schar Heart and Vascular Institute, Inova Fairfax Medical Campus, Falls Church, Virginia; b School of Medicine, University of Virginia, Charlottesville, Virginia; c Center for Cardiovascular Analytics, Research & Data Science, Providence-St. Joseph Health, Portland, Oregon; d Division of Cardiology, Beth Israel Deaconess Medical Center, Boston, Massachusetts; e Cardiovascular Institute at Allegheny Health Network, Pittsburgh, Pennsylvania; f Lifespan Cardiovascular Institute, Warren Alpert Medical School of Brown University, Providence, Rhode Island; g Division of Cardiology, Brigham & Women's Hospital, Harvard Medical School, Boston, Massachusetts; h Advanced Heart Disease Program, Baylor Scott & White Health, Temple, Texas; i Department of Cardiology, University of Oklahoma Health Science Center, Oklahoma City, Oklahoma; j Section of Cardiovascular Medicine, Department of Medicine, Wake Forest University School of Medicine, Winston-Salem, North Carolina; k The CardioVascular Center, Tufts Medical Center, Boston, Massachusetts

**Keywords:** devices, heart failure, medical management

## Abstract

Heart failure (HF) remains a major cause of morbidity and mortality worldwide. Major advancements in optimal guideline-directed medical therapy, including novel pharmacological agents, are now available for the treatment of chronic HF including HF with reduced ejection fraction and HF with preserved ejection fraction. Despite these efforts, there are several limitations of medical therapy including but not limited to: delays in implementation and/or initiation; inability to achieve target dosing; tolerability; adherence; and recurrent and chronic costs of care. A significant proportion of patients remain symptomatic with poor HF-related outcomes including rehospitalization, progression of disease, and mortality. Driven by these unmet clinical needs, there has been a significant growth of innovative device-based interventions across all HF phenotypes over the past several decades. This state-of-the-art review will summarize the current landscape of guideline-directed medical therapy for chronic HF, discuss its limitations including barriers to implementation, and review device-based therapies which have established efficacy or demonstrated promise in the management of chronic HF.

## Highlights

• Heart failure remains a major cause of morbidity and mortality worldwide.
• Medical therapies and device-based interventions for heart failure have evolved.
• We highlight both barriers to implementation and opportunities for improvement.

## Central Illustration

**Figure:**
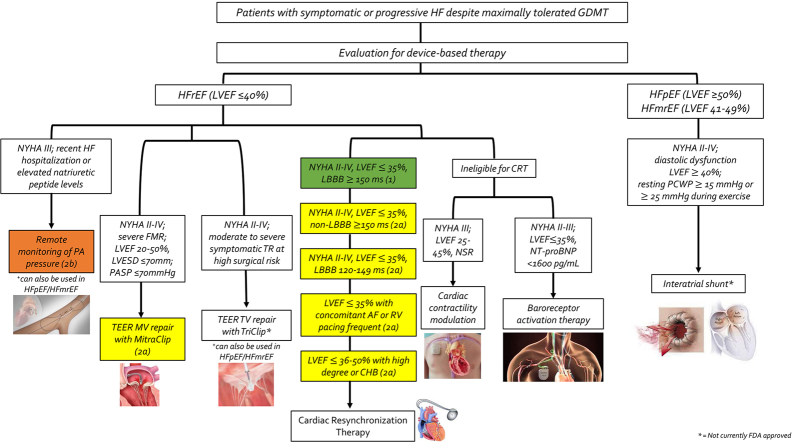

## Introduction

Heart failure (HF) is a complex clinical syndrome in which there is dyspnea or exertional limitation due to impairment of ventricular filling or ejection of blood or a combination of both.^1^ Despite multiple advances in the medical and device-based management of chronic HF, it remains a leading cause of morbidity and mortality globally and affects more than 6 million Americans and an estimated 23 million patients worldwide.^2^^,^^3^ Acute HF is the most common cause of hospitalization in older adults, with a 1-year hospitalization rate of 31.9% in patients with chronic HF.^2^^,^^3^ Chronic HF is currently classified according to the left ventricular ejection fraction (LVEF) into 3 phenotypes: heart failure with reduced ejection fraction (HFrEF, in which the LVEF is ≤40%); HF with midrange ejection fraction (HFmrEF, in which the LVEF is 41%-49%); and heart failure with preserved ejection fraction (HFpEF, in which the LVEF is ≥50%).^3^^,^^4^ The pathophysiology of HF is complex, as the deficiency of the heart to provide adequate perfusion leads to compensatory mechanisms including sympathetic activation, renal hypoperfusion with activation of the renin-angiotensin-aldosterone-system (RAAS), and stretching of the cardiac myocytes with subsequent release of natriuretic peptides, all of which initially serve to maintain adequate cardiac output.^5^ Over time, however, these responses lead to adverse cardiac remodeling characterized by left ventricular dilation, cardiac myocyte apoptosis, reduction in cardiac output and function, and further propagation of a vicious and progressive cycle of maladaptive responses.^5^^,^^6^

Over the past 4 decades, considerable progress has been made in the management of chronic HF with the use of optimal guideline-directed medical therapy (GDMT), which has been proven to dramatically reduce morbidity and mortality (Tables 1 and 2).^7^, ^8^, ^9^, ^10^, ^11^, ^12^, ^13^, ^14^, ^15^, ^16^, ^17^, ^18^, ^19^, ^20^, ^21^, ^22^, ^23^, ^24^, ^25^, ^26^, ^27^, ^28^, ^29^, ^30^, ^31^, ^32^ Unfortunately, the prevalence and health care expenditure of HF is expected to increase to greater than 8 million Americans and $69.7 billion USD by 2030, respectively.^33^ Driven by this unmet clinical need, novel device-based interventions have concurrently emerged as therapeutic options for patients with symptomatic HF across all phenotypes^34^ (Table 3).^35^, ^36^, ^37^, ^38^, ^39^, ^40^, ^41^, ^42^, ^43^, ^44^, ^45^, ^46^, ^47^, ^48^, ^49^ Several of these devices offer compelling advantages, including targeting specific pathophysiological pathways not amenable to drug therapy. This state-of-the-art review will summarize the current landscape of GDMT for chronic HF, discuss its limitations including barriers to implementation, and eliminating factors such as delays in implementation, achieving optimal dosing, adherence and tolerability, and chronic and recurrent costs of care and review the device-based interventions effective in the management of these patients.

**Table 1:** A comprehensive summary of the mainstay agents in the treatment of chronic HFrEF, evidenced by a conglomerate of landmark trials and the current clinical practice guideline recommendation.

Drug class	Mechanism of action	Physiologic response	Indication	Landmark RCTs	Pivotal evidence	2022 AHA/ACC/HFSA Class of Recommendation (COR) / Level of Evidence (LOE)
ARNI	Inhibition of angiotensin II receptor type 1 (AT1 receptor) [ARB component] + Inhibition of breakdown of natriuretic peptides [Neprilysin inhibitor component]	↑ Natriuresis, diuresis, and vasodilation ↓ Extracellular fluid ↓ NT-proBNP concentration ACEI/ARB effects as below	• All patients with chronic HFrEF and NYHA II-IV • Preferred over ACEI/ARB; can be started de novo	PARADIGM-HF^7^ PIONEER-HF^8^	Compared to ACEI: • Reduction in composite of CV death or first hospitalization for worsening HF (HR, 0.80; 95% CI, 0.73-0.87; *P* < .001)^7^ • Reduction in mortality (HR, 0.84; 95% CI, 0.76-0.93; *P* < .001)^7^ • Reduction in HFH (HR, 0.81; 95% CI, 0.71-0.89; *P* < .001)^7^ • Reduction in NT-proBNP concentration (Ratio of change 0.71 [95% CI, 0.63-0.81])^8^	COR 1 / LOE A
ACEI	Inhibition of ACE → ↓ conversion of angiotensin I to angiotensin II	↓ Vasoconstriction ↓ Aldosterone secretion → ↓ reabsorption of Na^+^ and water ↓ BP ↑ renal plasma flow → ↓ GFR → ↓ filtration fraction ↓ Afterload ↓ Preload Promote reverse cardiac remodeling	• All patients with chronic HFrEF, NYHA II-IV when ARNI is not feasible	CONSENSUS^9^ SOLVD^10^ SOLVD II^11^	• 40.0% reduction in mortality (52.0% vs 36.0%; *P* = .002)^9^ • Improvement in NYHA classification in ACEI group^9^ • 16.0% reduction in mortality (39.7% vs 35.2%; *P* = .004)^10^ • 26% reduction in death or HFH (*P* < .0001)^10^ • 36.0% reduction in HFH (18.7% vs 12.9%; P=.001)^11^	COR 1 / LOE A
ARB	Inhibition of angiotensin II receptor type 1 (AT1 receptor)		• All patients with chronic HFrEF, NYHA II-IV who are intolerant of ACEI due to cough or angioedema and when use of ARNI is not feasible	CHARM-Added^12^ Val-HeFT^13^	• Reduction in combined end point of CV death and HFH (HR, 0.85; 95% CI, 0.75-0.96)^12^ • 13.2% reduction in combined end point (morbidity & mortality, cardiac arrest, HFH, intravenous inotropic or vasodilator therapy) in ARB group vs placebo (HR, 0.87; 95% CI, 0.77-0.97)^13^	COR 1 / LOE A
BB (bisoprolol, metoprolol succinate, or carvedilol)	Competitively bind to and block β-adrenergic receptors	↓ BP ↓ Heart rate ↓ Cardiac contractility ↓ Myocardial O_2_ demand ↑ coronary perfusion Promote reverse cardiac remodeling	• All patients with chronic HFrEF • Should be continued even if asymptomatic or improving symptoms	CIBIS-II^14^ MERIT-HF^15^ COPERNICUS^16^	• Bisoprolol: 31% reduction in combined risk of death or HFH; reduced mortality (HR, 0.66; 95% CI, 0.54-0.81; *P* < .001); reduction in HFH (HR, 0.64; 95% CI, 0.53-0.79; *P* < .001)^14^ • Metoprolol succinate: 31% reduction in all-cause mortality (7.2% vs 11%; RR, 0.66; 95% CI, 0.53-0.81)^15^ • Carvedilol: 35% reduction in all-cause mortality (HR, 0.66; 95% CI, 0.54-0.81); 24.0% reduction in composite of HF death (12.0% vs 15.0%; *P* < .001)^16^	COR 1 / LOE A
MRA	Competitively bind to aldosterone receptors in the late distal convoluted tubule and the collecting duct	↓ Aldosterone effects → ↓ Na^+^ reabsorption and K^+^ excretion ↑ Diuresis Prevent adverse cardiac remodeling	• All patients with chronic HFrEF, NYHA II-IV, if eGFR >30 mL/min/1.73 m^2^ and serum potassium <5.0 mEq/L	RALES^17^ EMPHASIS-HF^18^	• 30.0% reduction in mortality (RR, 0.70; 95% CI, 0.60-0.82; *P* < .001)^17^ • 35.0% reduction in HF (RR, 0.65; 95% CI, 0.54-0.77; *P* < .001)^17^ • Improved NYHA class (*P* < .001)^17^ • Reduction in composite of mortality and HF (HR, 0.63; 95% CI, 0.54-0.74; *P* < .001)^18^ o Reduction in mortality (HR, 0.76; 95% CI, 0.61-0.93; *P* = .008)^18^ o Reduction in HF (HR, 0.58; 95% CI, 0.47-0.70; *P* < .001)^18^	COR 1 / LOE A
SGLT2i	Reversible inhibition of SGLT2 in the proximal tubule of the kidney	↑ Osmotic diuresis ↑ Natriuresis ↑ Glycosuria and polyuria ↓ BP Improve myocardial contractility	• All patients with symptomatic chronic HFrEF, irrespective of presence of T2DM	DAPA-HF^19^ EMPEROR-Reduced^20^ SOLOIST-WHF^21^	Reduction in primary composite end point of HFH or CV death: • HR, 0.74; 95% CI, 0.65-0.85; *P* < .001^19^ o HF events (HR, 0.70; 95% CI, 0.59-0.83)^19^ o CV death (HR, 0.82; 95% CI, 0.69-0.98)^19^ • HR, 0.75; 95% CI, 0.65-0.86; *P* < .001^20^ o HF events (HR, 0.70; 95% CI, 0.58-0.85)^20^ • Reduction in composite of CV death and HF events (HR, 0.67; 95% CI, 0.52-0.85; *P* < .001), benefit retained across HFrEF and HFpEF subgroups^21^	COR 1 / LOE A
Hydralazine-isosorbide dinitrate (H-ISDN)	Increase release of cGMP [Hydralazine] + Increase of nitric oxide [ISDN]	↑ Smooth muscle relaxation → ↑ vasodilation → ↓ preload ↓ Afterload ↓ BP ↓ Myocardial wall tension ↓ Myocardial O_2_ demand ↑ Myocardial perfusion	• Self-identified African Americans with NYHA III-IV HFrEF receiving optimal GDMT • In patients with symptomatic HFrEF who cannot be given first-line GDMT due to drug intolerance or renal insufficiency	V-HeFT I^22^ A-HeFT^23^	• 34% decrease in mortality at 2 y^22^ • 43% reduction in all-cause mortality^23^	COR 1 / LOE A: Self-identified African Americans COR 2b / LOE C-LD: Symptomatic HFrEF patients intolerant to first-line GDMT
Ivabradine	Selectively inhibits I_f_ channel in the pacemaker cells of the SA node → prolongs slow depolarization (phase 4)	↓ Heart rate ↓ Myocardial O_2_ demand ↑ Coronary perfusion Lengthen diastole	• All patients with stable chronic HFrEF, NYHA II-III who are receiving GDMT including a maximally tolerated BB and are in NSR with HR ≥70 bpm	SHIFT^24^	Composite end point of HFH or CV death: • HR, 0.82; 95% CI, 0.75-0.90; *P* < .0001^24^	COR 2a / LOR B-R
Soluble guanyl cyclase simulator (vericiguat)	Simulate soluble guanyl cyclase, increasing cGMP production	↑ Smooth muscle relaxation → ↑ vasodilation → ↓ preload ↑ Endothelial function ↓ Cardiac fibrosis Promote reverse cardiac remodeling	• Selected high-risk patients with HFrEF and recent worsening HF on optimal GDMT	VICTORIA^25^	Composite end point of HFH or CV death: • HR, 0.90; 95% CI, 0.82-0.98^25^	COR 2b /LOE B-R

ACEI, angiotensin-converting enzyme inhibitor; ARB, angiotensin II receptor blocker; ARNI, angiotensin receptor-neprilysin inhibitor; AT1, angiotensin II receptor type 1; BB, β-blocker; BP, blood pressure; CV, cardiovascular; eGFR, estimated glomerular filtration rate; GDMT, guideline-directed medical therapy; HF, heart failure; HFH, heart failure hospitalization; HFrEF, heart failure with reduced ejection fraction; HR, hazard ratio; MRA, mineralcorticoid receptor antagonist; NSR, normal sinus rhythm; NT-proBNP, N-terminal pro-brain natriuretic peptide; NYHA, New York Heart Association; RCT, randomized control trial; RR, relative risk; SGLT2i, sodium-glucose cotransporter 2 inhibitor; T2DM, type 2 diabetes mellitus.

**Table 2:** A comprehensive summary of the mainstay agents in the treatment of chronic HFpEF, evidenced by a conglomerate of landmark trials and current clinical practice guideline recommendation.

Drug class	Mechanism of action	Physiologic response	Indication	Pivotal evidence	2022 AHA/ACC/HFSA Class of Recommendation (COR) / Level of Evidence (LOE)
SGLT2i	Reversible inhibition of SGLT2 in the proximal tubule of the kidney	↑ Osmotic diuresis ↑ Natriuresis ↑ Glycosuria and polyuria ↓ BP Improve myocardial contractility	EMPEROR-Preserved^26^ DELIVER^27^	• Combined composite primary end point of CV death, HFH, or requiring IV treatment (HR, 0.77; 95% CI, 0.67-0.87)^26^ • HFH requiring intensive care (HR, 0.71; 95% CI, 0.52-0.96)^26^ • 20-50% improvement in NYHA functional class^26^ Composite end point of HFH or CV death: • HR, 0.79; 95% CI, 0.69-0.90; *P* < .001^27^	COR 2b / LOE B-R
MRA	Competitively bind to aldosterone receptors in the late distal convoluted tubule and the collecting duct	↓ Aldosterone effects → ↓ Na^+^ reabsorption and K^+^ excretion ↑ Diuresis Prevent adverse cardiac remodeling	Aldo-DHF^28^ TOPCAT^29^	• ↓ LV mass index (difference −6 g/m^2^; 95% CI, −10 to −1 g/m^2^)^28^ • Improved neuroendocrine activation (NT-proBNP geometric mean ratio 0.86; 95% CI, 0.75-0.99)^28^ • ⇔ HF symptoms or QoL^28^ • ↓ Incidence of HFH (HR 0.83 [95% CI 0.69-0.99])^29^ • ⇔ Total deaths or hospitalization for any reason^29^	COR 2b / LOE B-R
ARNI	Inhibition of angiotensin II receptor type 1 (AT1 receptor) [ARB component] + Inhibition of breakdown of natriuretic peptides [Neprilysin inhibitor component]	↑ Natriuresis, diuresis, and vasodilation ↓ Extracellular fluid ↓ NT-proBNP concentration ARB effects as below	PARAMOUNT-HF^30^ PARAGON-HF^31^	Compared to ARB: • ↓ NT-proBNP at 12 wk (ratio 0.77, 95% CI, 0.64-0.92; *P* = .005)^30^ • ⇔ CV death or HFH^31^ • Improvement in NYHA class (OR, 1.45; 95% CI, 1.13-1.86)^31^	COR 2b / LOE B-R
ARB	Inhibition of angiotensin II receptor type 1 (AT1 receptor)	↓ Vasoconstriction ↓ Aldosterone secretion → ↓ reabsorption of Na^+^ and water ↓ BP ↑ renal plasma flow → ↓ GFR → ↓ filtration fraction ↓ Afterload ↓ Preload Promote cardiac reverse remodeling	CHARM-Preserved^32^	• ⇔ Primary composite end point of CV death or HFH (HR, 0.89; 95% CI, 0.77-1.03)^32^ • ↓ Total no. of HFH and total hospitalizations in ARB arm vs control^32^	COR 2b / LOE B-R

ARB, angiotensin receptor blocker; ARNI, angiotensin receptor-neprilysin inhibitor; AT1, angiotensin II receptor type 1; BP, blood pressure; CV, cardiovascular; GFR, glomerular filtration rate; HFH, heart failure hospitalization; HFpEF, heart failure with preserved ejection fraction; HFrEF, heart failure with reduced ejection fraction; HR, hazard ratio; LV, left ventricular; MRA, mineralocorticoid receptor antagonist; NT-proBNP, N-terminal pro-brain natriuretic peptide; OR, odds ratio; QoL, quality of life; SGLT2i, sodium-glucose cotransporter 2 inhibitor.

## Medical management of chronic HFrEF

**Figure 1:**
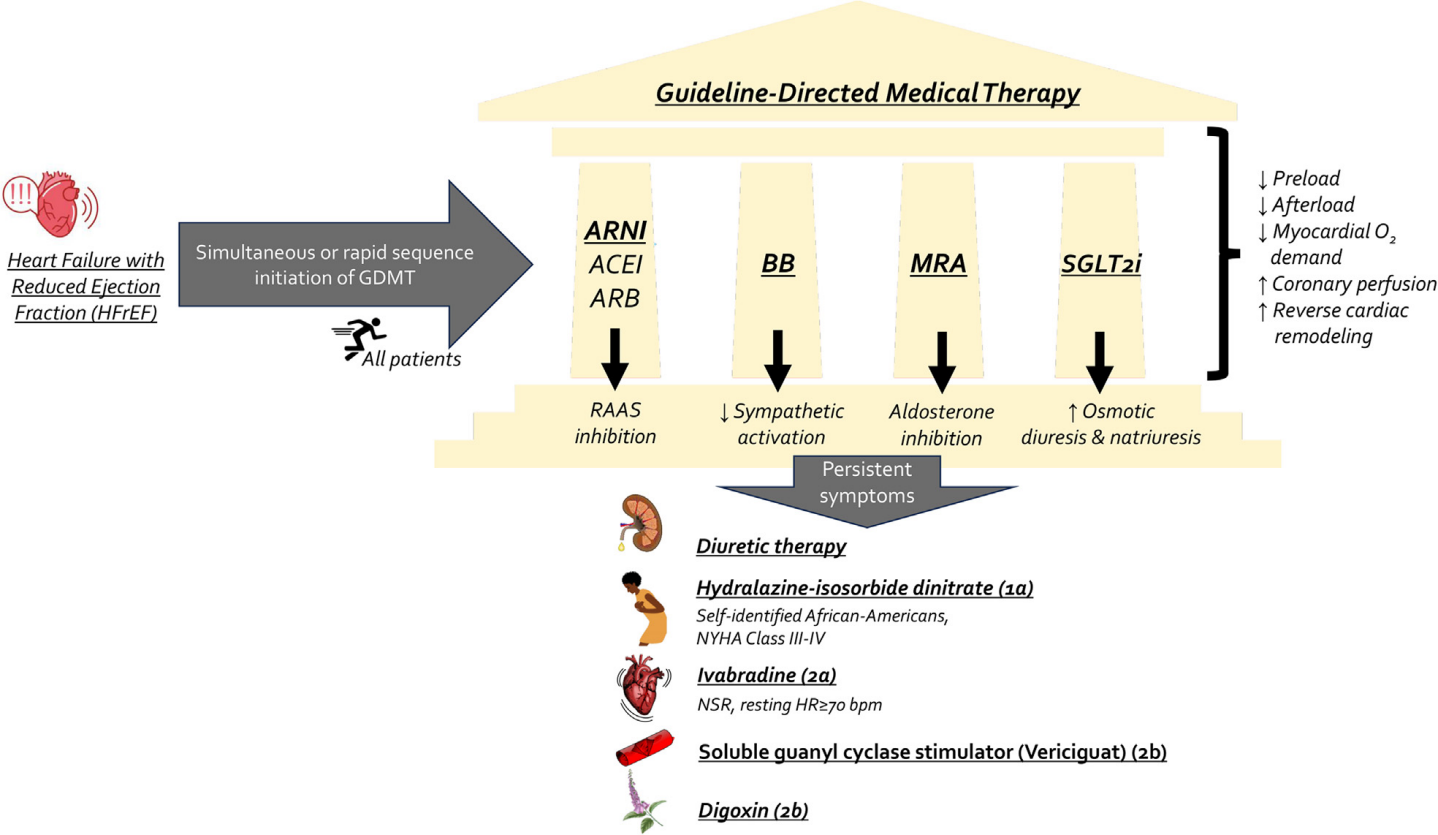
**Schema outlining the 4 mainstay guideline-directed medical therapies in the treatment of chronic heart failure with reduced ejection fraction (HFrEF) and additional treatment options for patients with persistent symptoms.** ACEI, angiotensin-converting enzyme inhibitor; ARB, angiotensin receptor blocker; ARNI, angiotensin receptor/neprilysin inhibitor; BB, β-blocker; GDMT, guideline-directed medical therapy; HR, heart rate; MRA, mineralocorticoid receptor antagonist; NSR, normal sinus rhythm; NYHA, New York Heart Association; RAAS, renin-angiotensin-aldosterone system; SGLT2i, sodium-glucose cotransporter-2 inhibitor.

Halting the deleterious and progressive cycle of HF has been the main therapeutic target of GDMT, improving cardiovascular (CV) outcomes and reversing detrimental cardiac remodeling for HFrEF patients.^6^ The 4 foundational pillars of pharmacologic GDMT have all shown significant reduction in morbidity and mortality (Figure 1): (1) angiotensin-converting enzyme inhibitors (ACEI)/angiotensin II receptor blockers (ARB)/angiotensin receptor-neprilysin inhibitors (ARNI), (2) β-blockers (BB), (3) mineralocorticoid receptor antagonists (MRA), and (4) sodium-glucose cotransporter 2 inhibitors (SGLT2i). A recent meta-analysis showed that the combination of ARNI, BB, MRA, and SGLT2i, as compared with conventional therapy, was most effective in reducing CV mortality and all-cause death and was estimated to extend the survival for a 55-year-old patient by 6.3 additional years and providing freedom from CV death or first hospitalization for HF by 8.3 additional years.^2^ Likewise, concurrent use of all 4 drug classes has been estimated to reduce all-cause mortality by 73%.^50^

By convention, the historical paradigm has been to prescribe GDMT in the specific sequence that randomized clinical trials used in testing them: namely, this involves starting with an ACEI/ARB followed by the add-on of a BB and then an MRA. If the patient remains symptomatic, an ARNI is introduced (typically switched from ACEI/ARB) before a SGLT2i is added. Furthermore, the doses of each therapy are increased to the guideline-recommended dosing (defined as target dose in the pivotal clinical trial) or the highest tolerated dose before initiating a new therapy. This traditional paradigm fails to recognize: (1) most of the landmark clinical trials did not involve patients who were already on optimized dosing of baseline HFrEF therapies at randomization; (2) the sequential algorithm may require 6 to 12 months to completely incorporate all the recommended therapies, leaving patients vulnerable to significant residual risk of CV morbidity and mortality; and (3) initiation of multiple therapies in concert, as opposed to sequentially, specifically ARNI and SGLT2i, may facilitate stabilization of potassium and renal function to enable initiation and tolerance of MRA.^51^ For these reasons, several authors have proposed simultaneous or rapid sequence initiation of GDMT (Figure 1).^52^

## ACEI, ARB, and ARNI

One of the hallmark maladaptive compensatory mechanisms in early HFrEF is RAAS activation resulting in vasoconstriction, fluid and salt retention, and systolic dysfunction promoting symptomatic HF. Pharmacologic inhibition of the RAAS pathway serves as the mainstay first-line therapeutic target for patients with HFrEF to reduce morbidity and mortality and promote reverse cardiac remodeling. Outcome data from the PARADIGM-HF (Prospective Comparison of ARNI with ACEI to Determine Impact on Global Mortality and Morbidity in Heart Failure) trial showed significant improvement in the composite primary end point of CV death or heart failure hospitalization (HFH) by 20% with use of the first approved ARNI, sacubitril-valsartan, relative to enalapril (ACEI).^7^ Further data from the PIONEER-HF (Comparison of Sacubitril/Valsartan versus Enalapril on Effect on NT-proBNP in Patients Stabilized from an Acute Heart Failure Episode) trial demonstrated that sacubitril-valsartan significantly reduced NT-pro-BNP levels and recurrent HFH compared to enalapril, without differences in hypotension, hyperkalemia, or acute kidney injury.^8^ Thus, in patients with New York Heart Association (NYHA) class II-III HFrEF, the use of ARNI is preferred over ACEI and ARB; in instances where ARNI use is not feasible, ACEI are preferred over ARB unless there is intolerance due to cough or angioedema (class 1 recommendation, level of evidence [LOE] A).^4^^,^^53^ Randomized controlled trial (RCT) data since PARADIGM-HF and PIONEER-HF demonstrate that de novo initiation of ARNI in ACEI/ARB-naïve patients has similar efficacy and safety outcomes without excess adverse events, compared to patients transitioned from previous ACEI/ARB therapy to ARNI, and can be initiated in patients hospitalized with acute HFrEF prior to discharge.^8^^,^^54^

## β-blockers

Consistent and compelling clinical trial evidence has shown that treatment with BB in HFrEF reduces HFH and all-cause mortality,^55^ with improvement in clinical status and LVEF.^14^, ^15^, ^16^^,^^56^, ^57^, ^58^ BB should be prescribed to all patients diagnosed with HFrEF, even when asymptomatic^59^ or when symptoms are mild or improve, as long-term BB therapy at target dose reduces the risk of progressive LV dysfunction and major CV events.^60^^,^^61^ Only 3 BB, in particular, have shown to be effective in the management of HFrEF: bisoprolol, sustained-release metoprolol (succinate), and carvedilol.^14^, ^15^, ^16^ Thus, use of 1 of these 3 agents should be initiated to reduce mortality and hospitalization in patients with HFrEF (class 1 recommendation, LOE A).^4^ The dose-response relationship is greatest with BBs and thus attempts should be made to optimize BB dosing to target or maximal doses that can be safely tolerated.^56^^,^^62^ BB therapy should not be discontinued even in cases of hospital admission, as data from the OPTIMIZE-HF (Organized Program to Initiate Lifesaving Treatment in Hospitalized Patients With Heart Failure) registry showed that BB discontinuation was associated with greater adjusted mortality risk postdischarge as compared to continuation.^63^^,^^64^ Furthermore, TRED-HF (Therapy withdrawal in REcovered Dilated cardiomyopathy – Heart Failure), an open-label, pilot RCT that demonstrated that many patients deemed to have recovered from dilated cardiomyopathy will relapse following treatment withdrawal, provides further evidence to support treatment continuation in chronic HFrEF patients.^60^

## MRA

MRA (ie, spironolactone or eplerenone), also known as aldosterone antagonists, have shown consistent benefit in reducing all-cause mortality, HFH, and sudden cardiac death in the management of chronic HFrEF.^17^^,^^18^^,^^65^ The effect of MRA therapy across a wide spectrum of HFrEF, inclusive of etiology and disease severity, was demonstrated by several RCTs. The RALES (Randomized Aldactone Evaluation Study) trial randomized symptomatic patients with a LVEF ≤35% and showed 30% reduction in all-cause mortality and 35% reduction in HFH.^17^ EPHESUS (Eplerenone Post-Acute Myocardial Infarction Heart Failure Efficacy and Survival Study) randomized post-acute myocardial infarction patients with a LVEF ≤40% and showed 15% reduction in all-cause mortality and 13% reduction in CV death and CV hospitalization.^65^ EMPHASIS-HF (Eplerenone in Mild Patients Hospitalization and Survival Study in Heart Failure) randomized patients with mild symptoms hospitalized with a LVEF ≤35% and showed 24% reduction in all-cause mortality and 37% reduction in CV death and HFH.^18^ MRA are partially excreted through the kidneys and can decrease renal potassium excretion, thus close monitoring of serum potassium and renal function should be performed at initiation and thereafter. An estimated glomerular filtration rate ≤30 mL/min/1.73 m^2^ or a serum potassium ≥5.0 mEq/L are US Food and Drug Administration (FDA) contraindications to MRA initiation.^4^ Accordingly, in all patients with chronic symptomatic HFrEF (namely, NYHA class II-IV) with an estimated glomerular filtration rate >30 mL/min/1.73 m^2^ and a serum potassium of <5.0 mEq/L, initiation of an MRA is recommended to reduce morbidity and mortality (class 1 recommendation, LOE A).^4^

## SGLT2i

The current guideline recommends that all patients with chronic symptomatic HFrEF be initiated on SGLT2i (ie, dapagliflozin or empagliflozin) therapy to reduce HFH and CV mortality, irrespective of history of type 2 diabetes (class 1 recommendation, LOE A).^4^^,^^19^^,^^20^ This recommendation is based on outcome data from 2 large RCTs, namely DAPA-HF (Dapagliflozin and Prevention of Adverse Outcomes in Heart Failure)^19^ and EMPEROR-Reduced (EMPagliflozin outcomE tRial in Patients With chrOnic heaRt Failure With Reduced Ejection Fraction)^20^.

DAPA-HF was a phase 3, placebo-controlled trial in which 4744 patients with NYHA class II-IV HFrEF received either dapagliflozin (at a dose of 10 mg once daily) or placebo, in addition to recommended therapy.^19^ Over a median of 18.2 months, the primary outcome, a composite of worsening HF (hospitalization or an urgent visit resulting in intravenous therapy for HF) or CV death, occurred in 16.3% in the dapagliflozin group and 21.2% in the placebo group (hazard ratio [HR], 0.74; 95% CI, 0.65-0.85; *P* < .001).^19^ A first worsening HF event occurred in 10.0% in the dapagliflozin group and 13.7% in the placebo group (HR, 0.70; 95% CI, 0.59-0.83).^19^ Death from CV causes occurred in 9.6% in the dapagliflozin group and 11.5% in the placebo group (HR, 0.82; 95% CI, 0.69-0.98); 11.6% and 13.9%, respectively, died from any cause (HR, 0.83; 95% CI, 0.71-0.97).^19^

EMPEROR-Reduced was a double-blinded RCT in which 3730 patients with NYHA class II-IV HFrEF received empagliflozin (at a dose of 10 mg once daily) or placebo, in addition to recommended therapy.^20^ During a median of 16 months, a primary outcome event, a composite of CV death or hospitalization for worsening HF, occurred in 19.4% in the empagliflozin group and 24.7% in the placebo group (HR, 0.75; 95% CI, 0.65-0.86; *P* < .001).^20^ The effect of empagliflozin on the primary outcome was consistent in patients regardless of the presence or absence of diabetes.^20^

### Adjunctive medical therapies for chronic HFrEF

#### Ivabradine

Ivabradine, a sinoatrial node modulator that selectively inhibits the hyperpolarization-activated cyclic-nucleotide gated funny (I_f_) current, causes prolongation of the slow depolarization phase of pacemaker cells (stage IV) and leads to a significant reduction in heart rate. Reduction in heart rate thereby reduces myocardial oxygen demand and increases the length of diastole, which can improve coronary perfusion. The SHIFT (Ivabradine and Outcomes in Chronic Heart Failure) trial tested this hypothesis and demonstrated a reduction in composite end point of HFH and CV death with use of ivabradine in patients with HFrEF.^24^ SHIFT included patients with symptomatic HFrEF (NYHA class II-III) with a LVEF ≤35% who had been hospitalized for HF in the preceding 12 months, with normal sinus rhythm (NSR) and resting heart rate ≥70 bpm.^24^ Participants were on stable GDMT for at least 4 weeks before initiation of ivabradine therapy; however, only 25% of patients studied were on optimal doses of BB therapy.^24^ Thus, given the established mortality benefits of BB therapy, current guidelines state that use of ivabradine can be beneficial for chronic HFrEF (LVEF ≤35%) patients in NSR with a resting heart rate of ≥70 bpm who remain symptomatic (NYHA class II-III) despite receiving optimal GDMT including a BB at a maximum tolerated dose (class 2a recommendation, LOE B-R).^4^

#### Combination hydralazine-isosorbide dinitrate (H-ISDN)

The benefit of combination H-ISDN in improving symptoms and reducing morbidity and mortality was established from 2 large consecutive RCTs—V-HeFT I (Vasodilator Heart Failure Trial)^22^ and A-HeFT (African-American Heart Failure Trial).^23^ The V-HeFT I trial was a multicenter, double-blinded, parallel-group RCT that randomized 642 patients at 11 Veterans Affairs centers in the United States with symptomatic chronic compensated systolic HFrEF from 1980 to 1985 to combination H-ISDN, prazosin, or placebo.^22^ At a mean follow-up of 2.3 years, there was no statistically significant difference in mortality between the 3 groups (38.7% vs 49.7% vs 44.0%, respectively).^22^ However, a post hoc subgroup analysis suggested improved survival with H-ISDN among self-identified African-American patients, thereby preempting the A-HeFT trial. Outcome data from A-HeFT, a multicenter, double-blinded, parallel-group, RCT conducted in 1050 patients at 61 centers in the United States from 2001 to 2004 limited its study to self-identified African-American patients.^23^ A-HeFT randomized patients to a fixed-dose H-ISDN or placebo in addition to standard GDMT with background therapy including an ACEI/ARB, BB, and MRA.^23^ The study was terminated early owing to a significantly higher mortality rate in the placebo group than in the H-ISDN group (10.2% vs 6.2%, *P* = .02), consistent with a nearly 40% reduction in all-cause mortality.^23^ Thus, current guidelines recommend combination H-ISDN for self-identified African-American patients with NYHA class III-IV symptoms with HFrEF despite use of GDMT with ACEI/ARB, BB, and MRA (class 1 recommendation, LOE A).^4^ There is insufficient data studying the concomitant use of H-ISDN with ARNI and/or SGLT2i therapy, suggesting the need for further high-quality studies. For patients with symptomatic HFrEF who are intolerant to first-line agents (eg, ARNI/ACEI/ARB) or those with renal insufficiency, referral to a HF specialist and use of H-ISDN as a therapeutic option can be considered (class 2b recommendation, LOE C-LD).^4^

#### Soluble guanylyl cyclase simulators

Novel therapeutic agents including oral soluble guanylyl cyclase stimulators (ie, vericiguat) have shown promise in benefiting patients with progressive HFrEF despite optimal GDMT. The mechanism involves direct binding and stimulation of soluble guanylyl cyclase leading to increased cyclic guanosine monophosphate production. Downstream effects of increased cyclic guanosine monophosphate lead to physiologic responses which can be advantageous for patients with refractory HF including vasodilation, augmentation of endothelial function, reverse cardiac remodeling, and decrease in cardiac fibrosis.^66^, ^67^, ^68^, ^69^, ^70^, ^71^ The VICTORIA (Vericiguat Global Study in Subjects with Heart Failure with Reduced Ejection Fraction) trial randomized patients with worsening HFrEF to vericiguat versus placebo, demonstrating reduction in the composite primary outcome of CV death and HFH in the vericiguat group vs placebo.^25^ Participants in VICTORIA had HFrEF with a LVEF < 45%, elevated natriuretic peptides, and NYHA II-IV symptoms with recent worsening HF (hospitalized within 6 months or had recently received intravenous diuretic therapy) on GDMT.^25^ Thus, current guidelines recommend consideration of vericiguat to reduce morbidity and mortality in select high-risk patients with worsening HFrEF despite optimal GDMT (class 2b recommendation, LOE B-R).^4^

#### Digoxin

Digoxin, a cardiac glycoside derived from the foxglove plant, is one of the oldest cardiac medications still in contemporary use.^72^ The effect of digoxin on reducing hospitalizations in a subset of patients with symptomatic HFrEF has been supported by both retrospective observational data and meta-analyses.^72^, ^73^, ^74^, ^75^ The pivotal DIG (Digitalis Investigation Group) trial demonstrated that patients randomized to the digoxin group versus placebo had lower rates of all-cause and HF-specific hospitalizations (relative risk, 0.72; 95% CI, 0.66-0.79; *P* < .001), without an effect on health-related quality of life (QoL) or mortality.^76^ Subsequent post hoc analysis of the DIG trial, however, demonstrated that compared to placebo, a serum digoxin concentration (SDC) of 0.5 to 0.9 ng/mL was associated with lower mortality (aHR, 0.85; 95% CI, 0.78-0.92) and HFH (aHR, 0.62; 95% CI, 0.54-0.72).^72^ Consistent with clinical guidelines, digoxin is usually initiated and maintained at a lower dose (eg, 0.125-0.25 mg daily) to achieve an SDC of 0.5 to 0.9 ng/mL given the detrimental effects higher SDC may have, including an independent linear relationship with mortality and worsening clinical deterioration after withdrawal.^77^, ^78^, ^79^ In patients with HFrEF who remain symptomatic despite optimal GDMT, digoxin may be considered to decrease HF-related hospitalization (class 2b recommendation, LOE B-R).^4^

## Medical management of chronic HFpEF

The diagnosis and treatment of HFpEF remains challenging largely due to its heterogeneity, including cardiac causes (eg, coronary artery disease, valvular dysfunction, hypertrophic cardiomyopathy, infiltrative disease, arrhythmias) and non-cardiac comorbidities including chronic kidney disease, diabetes, lung disease, and obesity.^80^, ^81^, ^82^ Current guidelines for the treatment of HFpEF include blood pressure (BP) control (class 1 recommendation, LOE C-LD), treatment of atrial fibrillation if present (class 2a recommendation, LOE C-EO), and use of SGLT2i (class 2a recommendation, LOE B-R), MRA, ARB, and ARNI (class 2b recommendation, LOE B-R).^4^

The EMPEROR-Preserved (Empagliflozin Outcome Trial in Patients with Chronic Heart Failure with Preserved Ejection Fraction) trial demonstrated a significant reduction in time to HFH and CV mortality with use of empagliflozin in patients with a LVEF >40% and chronic HF symptoms.^26^ This effect was mainly related to a lower risk of HFH in the empagliflozin group.^26^ The effects of empagliflozin appeared consistent in patients with or without diabetes.^26^ The total number of hospitalizations for HF was lower in the empagliflozin group than in the placebo group (HR, 0.73; 95% CI, 0.61-0.88; *P* < .001).^26^ The DELIVER (Dapagliflozin Evaluation to Improve the Lives of Patients with Preserved Ejection Fraction Heart Failure) trial demonstrated that dapagliflozin reduced the combined risk of worsening HF or CV death among patients with HFmrEF or HFpEF (HR, 0.82; 95% CI, 0.73-0.92; *P* < .001).^27^ Total events and symptom burden were lower in the dapagliflozin group than in the placebo group.^27^ Results were similar among both patients with a LVEF of ≥60% and <60%, and in prespecified subgroups including patients with or without diabetes.^27^ Thus, SGLT2i therapy is now recommended in patients with chronic HFmrEF and HFpEF (class 2a recommendation, LOE B-R).^4^

The TOPCAT (Treatment of Preserved Cardiac Function Heart Failure with an Aldosterone Antagonist) trial investigated the effects of MRA therapy in HFpEF patients, reporting that treatment with spironolactone did not reduce primary composite outcomes including CV death and HFH.^29^ Though not statistically significant, a small reduction in HFH was demonstrated with post hoc analyses evaluating clinical trial enrollment in the Americas, suggesting a potential benefit in select patients with symptomatic HFpEF.^29^^,^^83^ Thus, current guidelines recommend consideration of MRA therapy to reduce hospitalizations in patients with HFpEF.^4^

Similar to TOPCAT, various RCTs investigating the role of RAAS inhibition in patients with HFpEF have not shown much benefit, thus further research is warranted. For example, in the CHARM-Preserved (Candesartan in Patients with Chronic HF and Preserved Left-ventricular Ejection Fraction) trial, which randomized patients with a LVEF >40% to an ARB or placebo, showed no statistical difference in primary end point (CV death or HFH) among the 2 groups.^32^ Likewise, in the PARAGON-HF (Prospective Comparison of Angiotensin Receptor Neprilysin Inhibitor With Angiotensin Receptor Blocker Global Outcomes in Heart Failure and Preserved Left Ventricular Ejection Fraction) trial, use of sacubitril-valsartan compared to ARB (valsartan) did not achieve significant reduction in the primary combined end point of CV death and total HFH at a median follow-up of 35 months (rate ratio, 0.87; 95% CI, 0.75-1.01).^31^ However, post hoc patient-level analyses combining trials of HFpEF and HFrEF showed that sacubitril-valsartan, as compared with valsartan or enalapril, respectively, reduced the risk of HFH or CV mortality at a LVEF up to approximately 45% in men and 60% in women. Thus, the 2022 AHA/ACC/HFSA guidelines for HF provide ARNI/ARB therapy the same level of recommendation as MRA therapy for patients with chronic HFpEF (class 2b recommendation, LOE B-R).^4^

## Limitations in the current medical management for chronic heart failure

HF-associated morbidity, mortality, health care costs, and rehospitalization rates remain substantial due to several limitations in current medical therapy.^84^ Several important limitations include but are not limited to: the absence or delay in GDMT implementation, lack of initiation of GDMT during or following acute hospitalization, inability to achieve target dosing, and tolerability, adherence, and recurrent and chronic costs of care concerns.

### Absence or delay in GDMT implementation

Real-world data from prospective registries unfortunately suggest that initiation of GDMT is often significantly delayed or absent, and optimal therapy is not prescribed in a large enough proportion of eligible patients.^51^ For example, the prospective CHAMP-HF (Change the Management of Patients with Heart Failure) registry demonstrated that 27%, 33%, and 67% of eligible patients with HFrEF were not prescribed ACEI/ARB/ARNI, BB, and MRA therapy, respectively.^85^ Only 1% of eligible patients were initiated on triple therapy and 86% of patients were not prescribed an ARNI despite no medical contraindication.^51^^,^^85^ Analysis of claims-based data demonstrate similar findings, showing that 42% and 45% of patients with HFrEF were not prescribed any GDMT or only monotherapy within 30 day and 1-year post-index hospitalization, respectively.^86^^,^^87^ Recent data from EVOLUTION HF (Utilization of Dapagliflozin and Other Guideline-Directed Medical Therapies in Heart Failure Patients: A Multinational Observational Study Based on Secondary Data), the largest and most contemporary study following patients after incident HFH, demonstrated that the proportion of HFrEF patients with concomitant use of all 4 pillars of GDMT at 3 months of discharge was only 1.5% in the United States.^88^ Despite the high risk of rehospitalization associated with significant health care costs following incident HFH, use of GDMT in the year postdischarge did not change significantly, highlighting the need for earlier and greater implementation of GDMT.^84^

The underutilization of medical therapy may be, in part, due to the traditional paradigm that pharmacologic management should always follow a rigid sequential format and one therapy should be titrated to a maximally tolerated target dose prior to initiation of additional agents.^51^ Acknowledgment of this limitation and the substantial combined survival benefit of comprehensive GDMT has led to recent suggestions to emphasize rapid initiation of multiple agents in parallel succession.^2^ The recent randomized STRONG-HF (Safety, Tolerability and Efficacy of Rapid Optimization, Helped by NT-proBNP testinG, of Heart Failure Therapies) study underlined the feasibility and benefit of rapid initiation and optimization of 3 concurrent GDMT agents versus usual care.^84^^,^^89^

The STRONG-HF was a multinational, open-label, parallel-group RCT that randomized hospitalized patients with acute HF not on optimal doses of GDMT in a 1:1 fashion to high intensity up-titration (n = 542) or usual care (n = 536).^89^ Participants in the high intensity group underwent rapid titration to recommended doses of RAAS antagonists, BB, and MRA within 2 weeks of discharge and were followed over 2 months with at least 4 outpatient visits for close monitoring.^89^ The primary outcome, first occurrence for all-cause death or HF readmission by day 180, for the high intensity group vs usual care group was 15.2% vs 23.3% (*P* = .0021).^89^ At 90 days, a higher proportion of patients in the high intensity group had been uptitrated to recommended doses with a significant reduction in HFH or all-cause death.^89^ The risk of serious adverse events was similar in both groups. The study was stopped early per DSMB recommendations because of greater than expected between-group differences.^89^ Note that STRONG-HF was initiated prior to the approval of SGLT2i for the treatment of HFrEF and further studies will be needed to corroborate these findings.

### Lack of initiation of GDMT during or following acute hospitalization

An in-hospital or prehospital discharge initiation strategy to implementing GDMT has shown therapeutic benefit in reducing morbidity and mortality. Data from the PIONEER-HF trial demonstrated a greater reduction in NT pro-BNP concentration from baseline and improved clinical outcomes at 4 to 8 weeks in patients randomized to the in-hospital initiation ARNI therapy arm, suggesting the benefit of routine ARNI initiation for patients with HFrEF hospitalized for acute decompensated HF.^8^ Similarly, outcomes from the EMPULSE (Empagliflozin in Patients Hospitalized for Acute Heart Failure) and SOLOIST-WHF (Effect of Sotagliflozin on Cardiovascular Events in Patients with Type 2 Diabetes Post Worsening Heart Failure) trials demonstrated significant clinical benefit with an in-hospital initiation strategy for SGLT2i therapy for patients hospitalized with acute HF and a reduction in CV death and HFH with a prehospital discharge SGLT2i initiation strategy, respectively.^21^^,^^90^

Despite burgeoning research emphasizing the benefit of an in-hospital or prehospital discharge initiation strategy for increasing initiation, titration, and continuation of GDMT, data has shown that relatively few patients are treated with the required foundational therapies upon discharge. For example, in the IMPACT-HF (Initiation Management Predischarge: Process for Assessment of Carvedilol Therapy in Heart Failure) trial, participants randomized to the usual care arm versus predischarge initiation had significantly lower rates of BB use at 60 days despite lack of difference in hospital length of stay or adverse events among the 2 groups.^55^ Likewise, in the Get With The Guidelines-Heart Failure (GWTG-HF) registry, it was observed that >75% of eligible patients with HFrEF were not initiated on ARNI or MRA therapy in the year following discharge when the decision to initiate therapy was deferred after hospitalization.^91^

### Lack of target dosing achievement of GDMT

In the management of HFrEF, very few patients receive target doses of GDMT despite being discharged on suboptimal doses or no GDMT following hospitalization.^92^^,^^93^ Research has shown that nearly half of patients with HFrEF have had no changes made to their GDMT regimen in the year after hospitalization despite frequent evaluations in the outpatient setting.^51^^,^^87^ From CHAMP-HF, dose increases of GDMT occurred in ≤10% of patients with HFrEF within 1 year of hospitalization, even though very few patients were on target doses of BB (28%), ACEI/ARB (17%), and ARNI (14%) therapy.^85^ Of the participants in CHAMP-HF eligible for all classes of medication, only 1% of patients were simultaneously receiving target doses.^85^ Several potential barriers may explain suboptimal dosing of GDMT, such as clinician concern over the efficacy and safety of specific agents and a lack of multidisciplinary health care structures and strategic GDMT planning.^84^

### Tolerability, adherence, and cost of optimal medical therapy

The implementation of GDMT comes at the expense of increased complexity, added cost, and tolerability and adherence concerns.^51^ Contemporary, real-world practice remains challenging in a diverse patient population with increasing age, frailty, and psychosocial comorbidities. For example, depression and social isolation are 2 important risk factors associated with poor self-care and HFH.^94^^,^^95^ Low health literacy is also associated with poor HF self-care and low socioeconomic status, attributing to both ability to be adherent to and afford optimal medical therapy.^96^ Likewise, management of an increasing aging patient population and patients with concomitant renal insufficiency require close monitoring of their ability to tolerate optimal medical therapy, as appropriate age- and renal-dose adjustments may be necessary for many of the mainstay treatments.

The affordability and chronic and recurrent costs of GDMT, particularly with ARNI and SGLT2i therapy, is an important barrierthat includes, but is not limited to, prior authorization, insurance approval, and high out-of-pocket costs.^97^ For example, the annual average 2018 Medicare cost for ARNI and SGLT2i combined therapy was $10,802, with out-of-pocket costs of $895 among patients with prescription plans.^97^ At the median income for senior households ($47,000), these out-of-pocket costs would consume nearly 5% of income.^97^ These remarkable statistics only encompass the cost of GDMT, without considering the additive cost of management of the many comorbidities which accompany chronic HF.

## The role of device-based therapies in the management of chronic HF

Device-based therapies for HF are a rapidly evolving field which has shown significant promise in addressing the unmet substantial HF-related morbidity and mortality (Central Illustration, Table 3).^98^ A favorable shift in the regulatory device landscape has promoted and accelerated innovation.^34^ In 2018, the US FDA authorized the Breakthrough Devices Program to improve access to innovative devices indicated for the treatment of life-threatening illnesses including HF.^34^ Since 2019, the new Medicare coverage pathway, Medicare Coverage of Innovative Technology, now guarantees 4 years of coverage to its beneficiaries for breakthrough devices.^34^ The novel device therapies in the management of chronic HFrEF which will be discussed in this review include remote monitoring, cardiovascular implantable electronic devices (CIED), cardiac contractility modulation (CCM), autonomic modulation therapy, and transcatheter interventions for mitral and tricuspid regurgitation. The role of interatrial shunts and left atrial decompression therapies will also be discussed for the management of chronic HFpEF. Additional or emerging technologies under development are beyond the scope of this review and have been reviewed recently.^34^^,^^99^^,^^100^

**Central Illustration:**
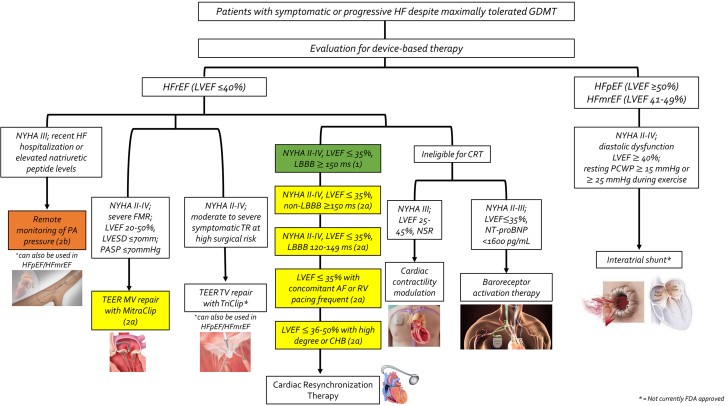
**Classification scheme outlining the indications for various device-based therapies in the management of chronic heart failure.** AF, atrial fibrillation; CHB, complete heart block; FMR, functional mitral regurgitation; GDMT, guideline-directed medical therapy; HF, heart failure; HFmrEF, heart failure with mildly reduced ejection fraction; HFpEF, heart failure with preserved ejection fraction; HFrEF, heart failure with reduced ejection fraction; LBBB, left bundle branch block; LVEF, left ventricular ejection fraction; LVESD, left ventricular end-systolic diameter; MV, mitral valve; NSR, normal sinus rhythm; NT-proBNP, N-terminal prohormone of brain natriuretic peptide; NYHA, New York Heart Association; PA, pulmonary artery; PASP, pulmonary artery systolic pressure; PCWP, pulmonary capillary wedge pressure; RV, right ventricle; TEER, transcatheter edge-to-edge repair; TR, tricuspid regurgitation; TV, tricuspid valve.

**Table 3:** A comprehensive summary of the current novel device-based therapies in the treatment of chronic heart failure.

Device	Mechanism	Response	Inclusion criteria	Pivotal evidence^a^	Clinical outcome	FDA approval
*Remote monitoring*						
CardioMEMS	Continuous pulmonary artery pressure monitoring	Detects intracardiac pressure before clinical decompensation	• HF hospitalization within 12 mo • Elevated BNP or NT-proBNP	CHAMPION^35^ GUIDE-HF^36^	↓ HFH ↓↓ HFH and mortality with concomitant optimal GDMT use	✓
Cordella				PROACTIVE-HF^a^	N/A	
*Cardiovascular implantable electronic devices*						
Cardiac resynchronization therapy (CRT)	Biventricular pacing	Decreases ventricular dyssynchrony Improves LV function Induces reverse ventricular remodeling	• NYHA II-IV, LVEF ≤ 35%, LBBB with QRS ≥ 150 ms • LVEF 36-50% with high degree or complete heart block • NYHA II-IV symptomatic HFrEF with non-LBBB pattern with QRS ≥ 150 ms • Genetic arrhythmogenic cardiomyopathy with high-risk features of SCD • High burden of ventricular pacing	MIRACLE^37^ COMPANION^38^ CARE-HF^39^ REVERSE^40^ MADIT-CRT^41^ RAFT^42^	↑ 6MWD, QoL, LVEF, LV remodeling ↓ NYHA class, HFH & mortality	✓
*Cardiac contractility modulation*						
OPTIMIZER Smart CE system	Delivery of nonexcitatory electrical signals to RV septal wall during absolute myocardial refractory period	Enhance myocardial contractility Augment myocardial calcium handling and gene expression	• NYHA III-IV, LVEF 25-45%, in NSR who remain symptomatic despite maximally tolerated GDMT and are CRT ineligible	FIX-HF-5C^43^ CCM-REG^44^	↑ Peak VO_2_, 6MWD, QoL ↓ NYHA class, HFH, & mortality	✓
*Autonomic modulation therapy*						
Barostim NEO System	Pulsed electrical stimulation of carotid sinus	Baroreceptor activation Reduction in sympathetic tone Increase in parasympathetic vagal tone	• NYHA II-III, LVEF ≤ 35% with NT-proBNP <1600 pg/mL who remain symptomatic despite maximally tolerated GDMT and are CRT ineligible	BeAT-HF^45^	↑ Functional status, QoL, 6MWD ↓ NT-proBNP MANCE free rate 97%	✓
Enopace (Harmony System)	Pulsed electrical stimulation of descending aorta		• Chronic HF with NYHA II-III symptoms on maximally tolerated GDMT	ENDO-HF^a^	N/A	
MobiusHD	Endovascular implant designed to reshape carotid sinus		• LVEF ≤ 40% with NYHA II-III symptoms despite maximally tolerated GDMT, NT-proBNP ≥ 400 pg/mL, 6MWD 150-400 m	HF-FIM^a^	N/A	
*Transcatheter interventions for valvular heart disease*						
MitraClip	Percutaneous, transcatheter, edge-to-edge repair via clip approximation of valve leaflets	Reduces severe functional mitral regurgitation	• Moderate-to-severe or severe symptomatic MR, LVEF 20-50% with NYHA II-IV despite optimal GDMT	COAPT^46^	↓ annualized HFH and all-cause mortality through 5 y follow-up ↓ Severity of FMR	✓
TriClip		Reduces severe functional tricuspid regurgitation	• Severe symptomatic TR with NYHA II-IV symptoms on stable GDMT ≥30 d, intermediate or greater risk of M&M with tricuspid valve surgery	TRILUMINATE^47^	↑ QoL ↓ Severity of TR ⇔ HFH or death MACE rate 1.7%	
*Implant-based interatrial shunt devices*						
IntraAtrial Shunt Device (IASD)	Device-based shunt implanted into the interatrial septum	Decrease left atrial pressure by providing pressure-dependent left-to-right atrial flow AFR: provides bidirectional atrial flow	• NYHA II-III symptoms, LVEF ≥ 40% with diastolic dysfunction, exercise PCWP ≥ 25 mm Hg while exceeding RAP ≥ 5 mm Hg	REDUCE-LAP-HF II^48^ RESPONDER-HF^a^	⇔ HFH or death	
V-wave shunt system			• NYHA II-IV symptoms with chronic HF, on maximally tolerated GDMT o If NYHA II → at least one HFH and BNP >300 pg/ml or NT-proBNP ≥ 1500 pg/mL o If NYHA III-IV → at least one HFH or BNP >300 pg/mL or NT-proBNP ≥ 1500 pg/mL	RELIEVE-HF^a^	↑ QoL (first 97 patients)	
Atrial Flow Regulator (AFR)			• NYHA III-IV symptomatic HFrEF & HFpEF patients with resting PCWP ≥15 mm Hg or ≥25 mm Hg during exercise	PRELIEVE^49^ FROST-HF^a^ AFteR^a^	↑ QoL, 6MWD ↓ NT-proBNP, PCWP, NYHA class	
APTURE Transcatheter Shunt System	Shunt implanted between left atrium and coronary sinus	Decrease left atrial pressure via left atrial to coronary sinus shunting	• NYHA II (with history of NYHA III) or NYHA III-IV symptoms and HFH or diuretic intensification or qualifying increased in BNP or NT-proBNP • On stable GDMT for 3 mo • Resting PCWP ≥15 mm Hg or ≥25 mm Hg during exercise	ALt FLOW US^a^	↑ QoL, health status ↓ NT-proBNP, PCWP, NYHA class	
*Implant-free interatrial shunt devices*						
Alleviant System	Radiofrequency ablation-based interatrial shunt (RAIAS) therapy to create an interatrial shunt without permanent implant placement	Decrease left atrial pressure by allowing for left-to-right atrial flow	• Symptomatic HFpEF/HFmrEF with LVEF ≥ 40 mm Hg, NYHA II-IV, exercise LAP ≥ 25 mm Hg, exercise PVR <1.8 WU	ALLAY-HF^a^	N/A	
NoYA System			• Chronic symptomatic HF with NYHA II-IV symptoms or HFH within the last year • On stable maximally tolerated GDMT • mLAP or resting PCWP ≥15 mm Hg, and mLAP or PCWP-RAP ≥5 mm Hg	NoYA RAISE Trial II^a^	N/A	

6MWD, 6 minute walk distance; BNP, B-type natriuretic peptide; FMR, functional mitral regurgitation; FDA, Food and Drug Administration; GDMT, goal-directed medical therapy; HF, heart failure; HFH, heart failure hospitalization; HFmrEF, heart failure with mildly reduced ejection fraction; HFpEF, heart failure with preserved ejection fraction; LAP, left atrial pressure; LBBB, left bundle branch block; LV, left ventricular; LVEF, left ventricular ejection fraction; MACE, major adverse cardiac events; MANCE, major adverse neurological and cardiovascular events; M&M, morbidity and mortality; MR, mitral regurgitation; N/A, not applicable; NSR, normal sinus rhythm; NT-proBNP, N-terminal pro-brain natriuretic peptide; NYHA, New York Heart Association; PCWP, pulmonary capillary wedge pressure; PVR, pulmonary vascular pressure; QoL, quality of life; RAP, right atrial pressure; RV, right ventricular; SCD, sudden cardiac death; TR, tricuspid regurgitation.

a Ongoing.

## Device-based therapies in the management of chronic HFrEF

### Remote monitoring

Continuous implantable pulmonary artery pressure (PAP) monitoring is a well-established device-based approach in remote monitoring which has shown significant reduction in HF-related hospitalization for symptomatic HF patients. Conceptually, this technology reflects the premise that an increase in intracardiac pressure typically precedes clinical or overt signs and symptoms of HF decompensation, and early detection of imminent congestion with invasive remote monitoring may prevent adverse CV outcomes.^35^^,^^101^ The CardioMEMS Heart Failure System (Abbott) is the only FDA-approved PAP monitoring system, consisting of a wirelessly implanted pulmonary artery sensor connected to an external electronic system via a secure internet database to store hemodynamic data that is readily available to the patient and care team.^35^^,^^102^ The CardioMEMS system was approved by the US FDA based on the results of the pivotal prospective, multicenter, open-label, single-blinded CHAMPION (CardioMEMS Heart Sensor Allows Monitoring of Pressure to Improve Outcomes in NYHA Class III Heart Failure Patients) trial, which randomized 550 patients with symptomatic NYHA class III HF with a recent HFH, all of whom received CardioMEMS device implantation, to PAP-guided therapy versus conventional care.^35^ Results from the CHAMPION trial showed significant reduction of 28% (HR, 0.72; 95% CI, 0.59–0.88) and 37% (HR, 0.63; 95% CI, 0.52-0.77) in HFH at 6 and 15 month follow-up, respectively, in the treatment group with use of CardioMEMS.^35^ Furthermore, a more pronounced reduction in all-cause mortality was appreciated for patients in the treatment group on mainstay GDMT with BB and ACEI/ARB/ARNI therapy (HR, 0.43; 95% CI, 0.26-0.76), suggesting a possible synergistic effect with use of optimal medical therapy and hemodynamic PAP monitoring with CardioMEMS.^102^^,^^103^ A comparable device, called the Cordella Pulmonary Artery Sensor System (Endotronix), is another innovative, completely remote PAP monitoring device seeking FDA approval which has similar hemodynamic principles to CardioMEMS though is combined with the Cordella Heart Failure System to provide additional real-time vital parameters including BP, heart rate, weight, and oxygen saturation. The prospective, multicenter, single-blinded PROACTIVE-HF (A Prospective, Multi-Center, Open Label, Single Arm Clinical Trial Evaluating the Safety and Efficacy of the Cordella Pulmonary Artery Sensor System in NYHA Class III Heart Failure Patients [NCT04089059]) trial recently completed enrollment in April 2023 and data evaluating the safety and effectiveness of this novel device is under review.^104^

### Cardiovascular implantable electronic devices (CIED)

CIED-based devices have evolved significantly and RCTs over the past 20 years have informed us on the role implantable cardioverter defibrillators play in the primary and secondary prevention of sudden cardiac death in patients with HFrEF (class 1 recommendation, LOE A).^4^^,^^105^ Cardiac resynchronization therapy (CRT), also known as biventricular pacing, is a device-based therapy that improves hemodynamics and LV function, reduces HFH, induces biological reverse remodeling,^6^ and improves QoL for patients with HFrEF.^105^ Current guideline recommendations for CRT come from seminal RCTs published from 2002-2010 including MIRACLE (Multicenter InSync Randomized Clinical Evaluation),^38^ COMPANION (Comparison of Medical Therapy, Pacing and Defibrillation in Heart Failure),^39^ CARE-HF (Cardiac Resynchronization Heart Failure),^40^ REVERSE (Resynchronization Reverses Remodeling in Systolic Left Ventricular Dysfunction),^41^ MADIT-CRT (Multicenter Automatic Defibrillator Implantation Trial-CRT),^42^ and RAFT (Resynchronization-Defibrillation for Ambulatory Heart Failure). Based on the amalgam of evidence from these studies, HFrEF patients who benefit most from CRT are those with symptomatic (NYHA class II-IV) HFrEF (LVEF ≤35%) with a concomitant left bundle branch block (LBBB) and wide QRS duration ≥150 ms (class 1 recommendation, LOE B-R).^4^ The benefits of CRT have also been extended to those with a LVEF between 36% and 50% with high degree or complete heart block,^106^^,^^107^ symptomatic HFrEF with a non-LBBB pattern with QRS duration ≥150 ms (class 2a recommendation, LOE B-R), as well as those with symptomatic HFrEF with LBBB with a QRS duration of 120-149 ms, concomitant atrial fibrillation, an expected high burden of ventricular pacing, and those with genetic arrhythmogenic cardiomyopathies with high-risk features of sudden cardiac death^108^^,^^109^ (class 2a recommendation, LOE B-NR).^4^

### Cardiac contractility modulation (CCM)

CCM is a device-based therapy that involves the delivery of high-voltage, nonexcitatory, electrical signals to the right ventricular septal wall during the absolute myocardial refractory period.^6^ CCM therapy has been shown to enhance myocardial contractility in the region of signal delivery^110^^,^^111^ due to augmentation of myocardial calcium handling^112^^,^^113^ by increasing calcium-induced calcium release from the sarcoplasmic reticulum and extracellular calcium influx.^34^ Chronic application of CCM induces favorable regulatory changes in the expression of several genes involved with intracellular calcium handling (which are adversely affected in chronic HF due to overactivation of the sympathetic nervous system and RAAS^114^) resulting in enhancement of global LV contractility.^110^^,^^111^ Outcome data from numerous RCTs have shown the clinical benefit of CCM therapy in patients with chronic symptomatic HFrEF, including improvement in functional status and QoL with a reduction in HFH.^113^

The OPTIMIZER Smart CE System (Impulse Dynamics), a CCM device-based therapy, received breakthrough device designation in 2015 and FDA approval in 2019 based on the results of the open-label FIX-HF-5C (Evaluate Safety and Efficacy of the OPTIMIZER System in Subjects With Moderate-to-Severe Heart Failure) trial for patients with NYHA class III-IV HFrEF with a LVEF between 25% and 45% in NSR who remain symptomatic despite GDMT and are ineligible for CRT.^43^^,^^115^ Randomizing 160 patients with NYHA class III-IV symptoms, LVEF 25% to 45%, and QRS duration <130 ms to either CCM or medical therapy, FIX-HF-5C demonstrated that patients randomized to the CCM arm had improved exercise capacity and QoL as measured by the MLWHFQ (Minnesota Living With HF Questionnaire), with reduced composite events of CV death and HFH.^43^^,^^115^ The recent prospective registry CCM-REG (Cardiac Contractility Modulation Registry) continues to provide long-term, real-world data on the contemporary impact of CCM therapy for 140 patients who were included using the same criterion as FIX-HF-5C, with an associated 75% decrease in HFH rate at 3 year follow-up.^44^

### Autonomic modulation therapy

Baroreceptors (stretch-sensitive nerve endings) are located in the walls of the carotid sinus, aortic arch, atria, and vena cava, which serve to detect BP in systemic circulation and play a fundamental role in the regulation of the autonomic nervous system (ANS). Better known as the baroreceptor reflex, these receptors sense changes in BP and send afferent signals to the vasomotor center of the medulla (solitary nucleus); subsequent efferent signals are transmitted to modulate sympathetic and parasympathetic tone to maintain BP homeostasis. ANS dysfunction with sympathetic overactivation and parasympathetic withdrawal is one of the hallmark maladaptive responses seen in HF^116^ causing accelerated CV stress and ventricular remodeling,^34^ and is a strong contributor to HF progression and mortality.^113^ Consequently, targeting this pathway to restore the sympathovagal physiological balance now serves as a therapeutic option for HF patients.

Baroreceptor activation therapy (BAT) leverages this intricate mechanism by delivering electrical impulses to the carotid sinus via an implanted device-based pulse generator.^117^^,^^118^ These impulses result in baroreceptor activation and manipulate the ANS, thereby causing a reduction in sympathetic outflow and an increase in parasympathetic vagal tone.^116^ Currently, the Barostim NEO system is the most widely used BAT device which was evaluated in the pivotal, prospective, multicenter, randomized BeAT-HF (Baroreflex Activation Therapy for Heart Failure) trial.^45^ Randomizing 408 patients with symptomatic (current or recent NYHA class III) HFrEF with a LVEF ≤35%, BeAT-HF demonstrated a significant improvement in functional status and MLWHFQ QoL scores, 6-minute walk distance (6MWD), and a reduction in NT-proBNP levels at 6 month follow-up in the patients randomized to BAT and medical therapy arm versus control (medical therapy alone).^45^ Based on these results, the Barostim NEO system received FDA approval in 2019 for patients with symptomatic (NYHA class II-III) HFrEF with a LVEF ≤35% despite maximally tolerated GDMT who are ineligible for CRT, with NT-proBNP levels <1600 pg/mL.^34^ Follow-up data from high-quality studies are needed to assess if the Barostim NEO system reduces long-term HF-related outcomes including HFH and mortality.^34^ Several other novel device-based therapies utilizing BAT technology have been developed and are currently undergoing clinical trials. The Enopace thoracic aortic stimulation system (Harmony implant), for example, is another BAT device which delivers electrical stimulation to the baroreceptors of the descending aorta resulting in baroreceptor activation and postulated effects including favorable left atrial remodeling and reduced LV wall stress.^119^^,^^120^ The ENDO-HF (Endovascular NeuromOdulation for Heart Failure [NCT02633644]) trial is an ongoing, open-label, single-arm study evaluating the safety and efficacy of Enopace thoracic aortic stimulation in patients with symptomatic HF.^120^ Preliminary results of ENDO-HF show promising results, with an improvement in exercise tolerance and MLWHFQ QoL scores and a reduction in NT-proBNP at 6 and 12 month follow-up.^120^ Also noteworthy is the MobiusHD system, an endovascular, device-based, BAT system which is implanted in the internal carotid artery and designed to reshape the carotid sinus to augment baroreceptor signaling and activation.^121^, ^122^, ^123^ The ongoing, open-label, single-arm HF-FIM (Effect of the MobiusHD in Patients With Heart Failure [NCT04590001]) trial aims to evaluate the safety and efficacy of the MobiusHD system in patients with symptomatic HFrEF.^122^

### Transcatheter interventions for mitral and tricuspid regurgitation

Functional (or secondary) mitral regurgitation (FMR) occurs frequently in patients with chronic HFrEF due to LV remodeling and dilation, which causes abnormal motion and impaired coaptation of the valve leaflets resulting in eccentric regurgitant jets.^6^^,^^46^ The prevalence of FMR continues to rise, with more than 4 million people in the USA expected to be diagnosed with FMR by 2030.^46^ Studies have shown that patients with significant FMR and HFrEF have an overall worse prognosis with a higher risk of CV mortality and HFH.^34^ While optimization of GDMT and use of CRT remain mainstay treatments for patients with severe FMR, many patients remain symptomatic with persistent FMR requiring evaluation for surgical intervention.^46^

Minimally invasive, percutaneous, transcatheter edge-to-edge repair (TEER) to correct FMR has gained significant momentum following the publication of the COAPT (Cardiovascular Outcomes Assessment of the MitraClip Percutaneous Therapy for Heart Failure Patients with Functional Mitral Regurgitation) trial.^46^ The MitraClip device, following TEER principles, creates a double orifice mitral valve by implementing a deliverable clip that approximates the A2/P2 mitral leaflets at the site of regurgitation.^46^ The 2 major RCTs, COAPT and MITRA-FR (Percutaneous Repair with the MitraClip Device for Severe Functional/Secondary Mitral Regurgitation), evaluating the survival benefit with the use of MitraClip in patients with FMR have resulted in considerable debate and controversy due to conflicting results. The multicenter MITRA-FR trial reported neutral results with no clinical benefit to MitraClip over GDMT in reducing HF for patients receiving optimal GDMT with severe, chronic, secondary MR.^6^^,^^124^ On the other hand, the landmark open-label, randomized COAPT trial demonstrated a significant reduction in HFH and all-cause mortality at 24 months in patients with persistent symptomatic HFrEF and severe FMR treated with MitraClip and optimal GDMT compared with optimal GDMT alone.^46^ Ultimately, the results of COAPT lead to the approval of MitraClip in 2019 in the US for FMR and should be considered for patients with symptomatic (NYHA class II-IV) HFrEF with a LVEF between 20% and 50% and moderate to severe FMR despite maximally tolerated GDMT and CRT if appropriate.^34^ Criticisms of the MITRA-FR trial mainly center around concerns that the patient population enrolled in the study were too sick to benefit from any intervention, as the cohort was noted to have higher rates of device failure and residual MR after MitraClip implementation, and higher rates of mortality and rehospitalization.^125^ Additionally, as compared to MITRA-FR, COAPT recruited patients with more severe MR, suggesting that patients with more progressive or disproportionate regurgitation are the ones who benefit most from MitraClip therapy.^6^ Certainly, further high-quality studies are warranted to elucidate the optimal patient population for selection of MitraClip implementation in treating severe persistent FMR.

Severe tricuspid regurgitation (TR) is also strongly associated with poor long-term survival and QoL.^126^ Treatment options for severe TR are limited, with medical therapy largely resolving around diuretic therapy and challenging surgical options posing high risks of complication.^47^ The TriClip (Abbott) device is a minimally invasive, TEER system, that has emerged as a safe and effective therapy for patients with severe TR that works similarly to the MitraClip device, possibly mitigating the need for cardiopulmonary bypass or cardiac surgery.^47^ Most noteworthy, the recent prospective landmark, multicenter TRILUMINATE (Trial to Evaluate Treatment With Abbott Transcatheter Clip Repair System in Patients With Moderate or Greater Tricuspid Regurgitation) Pivotal Trial was the first RCT to evaluate the effectiveness of TEER with the TriClip system compared to medical therapy alone for patients with severe TR and symptomatic HF.^47^ Outcome data from the TRILUMINATE Pivotal Trial showed that the TriClip system was safe and effective in the treatment of severe TR, demonstrating a significant improvement in QoL scores and a reduction in the severity of TR from severe to moderate or less in 87% of the patients randomized to the TriClip TEER arm versus 4.8% in the control arm at 30 days.^47^ This reduction remained stable at 1-year follow-up.^47^ Longitudinal studies will be crucial to determine if TriClip has an effect in reducing HFH and mortality given these notable clinical benefits.

## Device-based therapies in the management of chronic HFpEF

HFpEF is now the most common cause of HF within the United States, in part due to an aging patient population and increased incidence of comorbidities such as diabetes and obesity.^127^ The effective management of HFpEF remains a major unmet clinical need. Impaired compliance and LV relaxation are hallmark features of HFpEF, leading to elevated LV filling pressures and left atrial pressure (LAP).^127^ Elevated LAP at rest or during exercise is pathognomonic in HF and is a key determinant of pulmonary congestion, consequently leading to pulmonary hypertension, exertional dyspnea, and progression of HF due to adverse left atrial remodeling.^100^^,^^128^ Thus, a series of safe and effective device-based therapies to reduce LAP via an iatrogenic interatrial shunt approach, using an implant-based or implant-free system, have been developed which have shown promise in improving QoL, HF-related outcomes, and exercise tolerance.

### InterAtrial Shunt Device and V-Wave Shunt System

The InterAtrial Shunt Device (IASD, Corvia Medical) is an interatrial shunt system, consisting of a self-expanding bare-metal stent with a double-disc shape implanted into the atrial septum, allowing for physiological unidirectional pressure-dependent left-to-right atrial flow.^129^ Preliminary trials showed that the IASD decreased exercise pulmonary capillary wedge pressure (PCWP) without device-related complications or major adverse events.^101^^,^^129^ As a follow-up and the first phase 3 RCT investigating the IASD, the prospective, multicenter REDUCE-LAP-HF II (A Study to Evaluate the Corvia Medical, Inc. IASD System II to Reduce Elevated Left Atrial Pressure in Patients With Heart Failure) trial randomized 626 patients with a LVEF ≥40% and exercise PCWP ≥25 mm Hg to IASD or sham procedure.^48^ Recent 2-year follow-up outcome data showed no statistical difference between the 2 groups in both the overall and individual components of the primary composite end point (hierarchical composite of CV mortality or nonfatal ischemic stroke, HFH, intensification of diuretics, or change in health status).^130^ Further subgroup analysis of the patients who underwent IASD implantation showed that patients with exercise-induced left atrial hypertension (EILAH) were more likely to have characteristics associated with atrial shunt responsiveness as compared to patients with resting left atrial hypertension (RELAH).^130^ However, patients with EILAH were also shown to have less advanced myocardial disease and fewer coexisting comorbidities, underscoring the importance of invasive hemodynamic phenotyping for proper patient selection for device-based interventions.^130^ The ongoing prospective, double-blinded, sham-controlled RESPONDER-HF (Re-Evaluation of the Corvia Atrial Shunt Device in a Precision Medicine Trial to Determine Efficacy in Mildly Reduced or Preserved Ejection Fraction (EF) Heart Failure [NCT05425459]) trial is designed to confirm the safety and clinical efficacy of IASD implementation for symptomatic HF patients with a LVEF ≥40% and elevated left-sided filling pressures despite optimal GDMT.

The V-Wave Shunt System (Caesarea) is another interatrial shunt system that forms an hourglass shape across the septum and provides unidirectional left-to-right atrial flow, similar to the IASD.^34^ A first-in-human, multicenter, single-arm, open-label study demonstrated initial safety and early clinical benefit including improvement in NYHA functional class, PCWP, and QoL in patients with HF.^131^ However, a high rate of shunt stenosis and occlusion from valve-related pannus formation causing loss of efficacy in nearly half the cases was also observed, prompting development of the second iteration of the device eliminating its valve component.^128^ The ongoing RELIEVE-HF (REducing Lung congestIon Symptoms Using the v-wavE Shunt in adVancEd Heart Failure [NCT03499236]) trial will evaluate the safety and efficacy of the second-generation V-Wave Shunt in patients with symptomatic HF (NYHA III-IV), regardless of LVEF.^128^ Analysis of the first 97 patients show promising results, including a high rate of successful implementation and safety with sustained QoL improvement in the first month.^128^

### Atrial Flow Regulator (Occlutech)

The Atrial Flow Regulator (AFR, Occlutech) is an implant-based interatrial shunt approach that allows for bidirectional atrial flow, rather than unidirectional flow, due to its circular double-disc nitinol braided mesh design.^128^ The opening of the central fenestration is also available in various diameters, allowing for patient-specific selection based on PCWP and septal thickness.^127^ The first-in-human, open-label, prospective, multicenter PRELIEVE (Pilot Study to Assess Safety and Efficacy of a Novel Atrial Flow Regulator (AFR) in Heart Failure Patients) trial investigated the feasibility of AFR implementation in both HFrEF and HFpEF patients up to 1-year follow-up.^49^ Results of PRELIEVE demonstrated a reduction in 3-month resting PCWP among the whole cohort, with the PCWP change significant for HFpEF patients as compared to HFrEF patients.^49^ At 1-year follow-up, continued device patency with sufficient echocardiography was observed as well as an improvement in NYHA functional class, QoL, and 6MWD in individual patients from both HFrEF and HFpEF groups.^49^ Several studies including the FROST-HF (Flow Regulation by Opening the Septum in Patients With Heart Failure [NCT03751748]) trial and the large observational registry AFteR (Follow-up Study to Monitor the Efficacy and Safety of the Occlutech AFR in Heart Failure Patients [NCT04405583]) registry are underway and aim to further test the safety and efficacy of the AFR.

### Transcatheter Atrial Shunt System (Edwards)

Unique to the aforementioned devices above, which utilize the interatrial septum as its site of shunt placement, the Transcatheter Atrial Shunt System (TASS, Edwards Lifesciences) creates a shunt from the left atrium to the coronary sinus to reduce PCWP.^128^ This novel shunt approach was developed given suggested limitations with interatrial shunting, including elevated risk of right heart volume overload, hypoxemia, systemic embolization, and limiting necessary transseptal access for future percutaneous procedures.^128^ In the first-in-human study, the TASS device was successfully implanted in 8 of the 11 patients, with observed reduction in PCWP and improvement in Kansas City Cardiomyopathy Questionnaire (KCCQ) QoL score and NYHA functional class.^127^ The ongoing, prospective, feasibility study ALt FLOW US (EArLy FeasibiLity Study Of the EdWards Transcatheter Atrial Shunt System [NCT03523416]) will evaluate the safety and efficacy of the TASS device in a larger patient population.

### Alleviant System and NoYA system

Noteworthy and innovative implant-free, stentless systems have been developed including the Alleviant System and NoYa system (DiNoVa Medtech), both of which involve radiofrequency ablation-based interatrial shunt (RAIAS) therapy. The Alleviant System, which gained Investigational Device Exemption by the FDA in November 2022, is a minimally-invasive transcatheter device which utilizes RAIAS therapy to excise tissue to create a durable interatrial connection without leaving a permanent implant in situ.^132^ Outcome data at 6-month follow-up from early feasibility studies demonstrated favorable safety and efficacy of the Alleviant System.^132^ ALLAY-HF (Safety and Efficacy of the Alleviant System for No-Implant Interatrial Shunt Creation in Patients With Chronic Heart Failure [NCT05685303]) is a global prospective, multicenter, sham-controlled, double-blinded RCT which began enrollment in 2023 and will evaluate the safety and effectiveness of the Alleviant System for chronic HFmrEF and HFpEF patients who remain symptomatic despite optimal GDMT. ALLAY-HF plans to enroll 400 to 700 patients with composite primary end point analysis to be conducted at 1-year follow-up. Similar to the Alleviant System, the NoYa system is a novel nonimplantable device which uses RAIAS therapy, consisting of a self-expandable flowerlike stent which is diameter adjustable.^128^ Preliminary data from a first-in-human trial showed improvement of NYHA functional class and 6MWD with a reduction in NT-proBNP levels after RAIAS therapy with the NoYa system, though future prospective RCTs are warranted to elucidate its long-term safety and efficacy.^128^

## Conclusion and future directions

The recommendations for the effective treatment of chronic HFrEF and HFpEF continue to progress, with remarkable advancements in both optimal GDMT and device-based interventions. Therapeutic device-based interventions are becoming integral in the management of chronic HF and present a promising opportunity to address the associated morbidity and mortality. More randomized studies are needed before a paradigm shift occurs in implementing device-based therapies earlier in the treatment course, thereby replacing the standard conventional approach of optimizing drug therapy first prior to device therapy initiation.^133^ Indeed, devices may augment and facilitate optimization of medical therapy by improving hemodynamics and targeting some important pathophysiological pathways more effectively than drug therapy.^134^ However, identifying the ideal patient population for appropriate selection of device-based therapies has remained challenging, emphasizing the need for molecular, clinical, biochemical, and physiological phenotyping. Additionally, further studies should elucidate the synergies and disadvantages of multiple drug and device-based interactions, as many of the currently available therapies target similar patients who may be eligible for more than one device. Lastly, future prospective, high-quality, randomized controlled studies with hard clinical primary end points (eg, reduction in mortality) as well as patient-reported outcomes are needed to improve the care of this vulnerable patient population.

## References

[1] Murphy S.P., Ibrahim N.E., Januzzi J.L. (2020). Heart failure with reduced ejection fraction: a review. JAMA.

[2] Tromp J., Ouwerkerk W., Van Veldhuisen D.J. (2022). A systematic review and network meta-analysis of pharmacological treatment of heart failure with reduced ejection fraction. J Am Coll Cardiol HF.

[3] Rosenblum H., Kapur N.K., Abraham W.T. (2020). Conceptual considerations for device-based therapy in acute decompensated heart failure: DRI _2_ P _2_ S. Circ Heart Fail.

[4] Heidenreich P.A., Bozkurt B., Aguilar D. (2022). 2022 AHA/ACC/HFSA guideline for the management of heart failure: a report of the American College of Cardiology/American Heart Association Joint Committee on Clinical Practice Guidelines. Circulation.

[5] Koitabashi N., Kass D.A. (2011). Reverse remodeling in heart failure--mechanisms and therapeutic opportunities. Nat Rev Cardiol.

[6] Brener M.I., Uriel N., Burkhoff D. (2020). Left ventricular volume reduction and reshaping as a treatment option for heart failure. Struct Heart.

[7] McMurray J.J.V., Packer M., Desai A.S. (2014). Angiotensin–neprilysin inhibition versus enalapril in heart failure. N Engl J Med.

[8] Velazquez E.J., Morrow D.A., DeVore A.D. (2019). Angiotensin-neprilysin inhibition in acute decompensated heart failure. N Engl J Med.

[9] Consensus Trial Study Group (1987). Effects of enalapril on mortality in severe congestive heart failure. Results of the Cooperative North Scandinavian Enalapril Survival Study (CONSENSUS). N Engl J Med.

[10] Investigators S.O.L.V.D., Yusuf S., Pitt B., Davis C.E., Hood W.B., Cohn J.N. (1991). Effect of enalapril on survival in patients with reduced left ventricular ejection fractions and congestive heart failure. N Engl J Med.

[11] Investigators S.O.L.V.D., Yusuf S., Pitt B., Davis C.E., Hood W.B., Cohn J.N. (1992). Effect of enalapril on mortality and the development of heart failure in asymptomatic patients with reduced left ventricular ejection fractions. N Engl J Med.

[12] McMurray J.J., Östergren J., Swedberg K. (2003). Effects of candesartan in patients with chronic heart failure and reduced left-ventricular systolic function taking angiotensin-converting-enzyme inhibitors: the CHARM-Added trial. Lancet.

[13] Cohn J.N., Tognoni G. (2001). Valsartan Heart Failure Trial Investigators. A randomized trial of the angiotensin-receptor blocker valsartan in chronic heart failure. N Engl J Med.

[14] Cardiac Insufficiency Authors (1999). The Cardiac Insufficiency Bisoprolol Study II (CIBIS-II): a randomised trial. Lancet.

[15] (1999). Effect of metoprolol CR/XL in chronic heart failure: Metoprolol CR/XL Randomised Intervention Trial in-Congestive Heart Failure (MERIT-HF). Lancet.

[16] Packer M., Fowler M.B., Roecker E.B. (2002). Effect of carvedilol on the morbidity of patients with severe chronic heart failure: results of the Carvedilol Prospective Randomized Cumulative Survival (COPERNICUS) study. Circulation.

[17] Pitt B., Zannad F., Remme W.J. (1999). The effect of spironolactone on morbidity and mortality in patients with severe heart failure. N Engl J Med.

[18] Zannad F., McMurray J.J.V., Krum H. (2011). Eplerenone in patients with systolic heart failure and mild symptoms. N Engl J Med.

[19] McMurray J.J.V., Solomon S.D., Inzucchi S.E. (2019). Dapagliflozin in patients with heart failure and reduced ejection fraction. N Engl J Med.

[20] Packer M., Anker S.D., Butler J. (2020). Cardiovascular and renal outcomes with empagliflozin in heart failure. N Engl J Med.

[21] Bhatt D.L., Szarek M., Steg P.G. (2021). Sotagliflozin in patients with diabetes and recent worsening heart failure. N Engl J Med.

[22] Cohn J.N., Archibald D.G., Ziesche S. (1986). Effect of vasodilator therapy on mortality in chronic congestive heart failure. Results of a Veterans Administration Cooperative Study. N Engl J Med.

[23] Taylor A.L., Ziesche S., Yancy C. (2004). Combination of isosorbide dinitrate and hydralazine in blacks with heart failure. N Engl J Med.

[24] Swedberg K., Komajda M., Böhm M. (2010). Ivabradine and outcomes in chronic heart failure (SHIFT): a randomised placebo-controlled study. Lancet.

[25] Armstrong P.W., Pieske B., Anstrom K.J. (2020). Vericiguat in patients with heart failure and reduced ejection fraction. N Engl J Med.

[26] Anker S.D., Butler J., Filippatos G. (2021). Empagliflozin in heart failure with a preserved ejection fraction. N Engl J Med.

[27] Solomon S.D., McMurray J.J.V., Claggett B. (2022). Dapagliflozin in heart failure with mildly reduced or preserved ejection fraction. N Engl J Med.

[28] Edelmann F., Wachter R., Schmidt A.G. (2013). Effect of spironolactone on diastolic function and exercise capacity in patients with heart failure with preserved ejection fraction: the Aldo-DHF randomized controlled trial. JAMA.

[29] Pitt B., Pfeffer M.A., Assmann S.F. (2014). Spironolactone for heart failure with preserved ejection fraction. N Engl J Med.

[30] Solomon S.D., Zile M., Pieske B. (2012). The angiotensin receptor neprilysin inhibitor LCZ696 in heart failure with preserved ejection fraction: a phase 2 double-blind randomised controlled trial. Lancet.

[31] Solomon S.D., McMurray J.J.V., Anand I.S. (2019). Angiotensin–neprilysin inhibition in heart failure with preserved ejection fraction. N Engl J Med.

[32] Yusuf S., Pfeffer M.A., Swedberg K. (2003). Effects of candesartan in patients with chronic heart failure and preserved left-ventricular ejection fraction: the CHARM-Preserved Trial. Lancet.

[33] Jackson S.L., Tong X., King R.J., Loustalot F., Hong Y., Ritchey M.D. (2018). National burden of heart failure events in the United States, 2006 to 2014. Circ Heart Fail.

[34] Fudim M., Abraham W.T., von Bardeleben R.S. (2021). Device therapy in chronic heart failure: JACC state-of-the-art review. J Am Coll Cardiol.

[35] Abraham W.T., Adamson P.B., Bourge R.C. (2011). Wireless pulmonary artery haemodynamic monitoring in chronic heart failure: a randomised controlled trial. Lancet.

[36] Lindenfeld J, Zile MR, Desai AS (2021). Haemodynamic-guided management of heart failure (GUIDE-HF): a randomised controlled trial. Lancet.

[37] Abraham W.T., Fisher W.G., Smith A.L. (2002). Cardiac resynchronization in chronic heart failure. N Engl J Med.

[38] Bristow M.R., Saxon L.A., Boehmer J. (2004). Cardiac-resynchronization therapy with or without an implantable defibrillator in advanced chronic heart failure. N Engl J Med.

[39] Cleland J.G.F., Daubert J.C., Erdmann E. (2005). The effect of cardiac resynchronization on morbidity and mortality in heart failure. N Engl J Med.

[40] Linde C., Abraham W.T., Gold M.R. (2008). Randomized trial of cardiac resynchronization in mildly symptomatic heart failure patients and in asymptomatic patients with left ventricular dysfunction and previous heart failure symptoms. J Am Coll Cardiol.

[41] Moss A.J., Hall W.J., Cannom D.S. (2009). Cardiac-resynchronization therapy for the prevention of heart-failure events. N Engl J Med.

[42] Tang A.S.L., Wells G.A., Talajic M. (2010). Cardiac-resynchronization therapy for mild-to-moderate heart failure. N Engl J Med.

[43] Abraham W.T., Kuck K.H., Goldsmith R.L. (2018). A randomized controlled trial to evaluate the safety and efficacy of cardiac contractility modulation. J Am Coll Cardiol HF.

[44] Anker S.D., Borggrefe M., Neuser H. (2019). Cardiac contractility modulation improves long-term survival and hospitalizations in heart failure with reduced ejection fraction. Eur J Heart Fail.

[45] Zile M.R., Lindenfeld J., Weaver F.A. (2020). Baroreflex activation therapy in patients with heart failure with reduced ejection fraction. J Am Coll Cardiol.

[46] Stone G.W., Lindenfeld J., Abraham W.T. (2018). Transcatheter mitral-valve repair in patients with heart failure. N Engl J Med.

[47] Sorajja P., Whisenant B., Hamid N. (2023). Transcatheter repair for patients with tricuspid regurgitation. N Engl J Med.

[48] Shah S.J., Borlaug B.A., Chung E.S. (2022). Atrial shunt device for heart failure with preserved and mildly reduced ejection fraction (REDUCE LAP-HF II): a randomised, multicentre, blinded, sham-controlled trial. Lancet.

[49] Paitazoglou C., Özdemir R., Pfister R. (2019). The AFR-PRELIEVE trial: a prospective, non-randomised, pilot study to assess the Atrial Flow Regulator (AFR) in heart failure patients with either preserved or reduced ejection fraction. EuroIntervention.

[50] Bassi N.S., Ziaeian B., Yancy C.W., Fonarow G.C. (2020). Association of optimal implementation of sodium-glucose cotransporter 2 inhibitor therapy with outcome for patients with heart failure: how do we translate these findings into clinical care?. JAMA Cardiol.

[51] Sharma A., Verma S., Bhatt D.L. (2022). Optimizing foundational therapies in patients with HFrEF. J Am Coll Cardiol Basic Trans Science.

[52] Greene S.J., Butler J., Fonarow G.C. (2021). Simultaneous or rapid sequence initiation of quadruple medical therapy for heart failure-optimizing therapy with the need for speed. JAMA Cardiol.

[53] Belkin M.N., Cifu A.S., Pinney S. (2022). Management of heart failure. JAMA.

[54] Wachter R., Senni M., Belohlavek J. (2019). Initiation of sacubitril/valsartan in haemodynamically stabilised heart failure patients in hospital or early after discharge: primary results of the randomised TRANSITION study. Eur J Heart Fail.

[55] Gattis W.A., O’Connor C.M., Gallup D.S., Hasselblad V., Gheorghiade M. (2004). IMPACT-HF Investigators and Coordinators. Predischarge initiation of carvedilol in patients hospitalized for decompensated heart failure: results of the Initiation Management Predischarge: Process for Assessment of Carvedilol Therapy in Heart Failure (IMPACT-HF) trial. J Am Coll Cardiol.

[56] Bristow M.R., Gilbert E.M., Abraham W.T. (1996). Carvedilol produces dose-related improvements in left ventricular function and survival in subjects with chronic heart failure. Circulation.

[57] Cleland J.G.F., Bunting K.V., Flather M.D. (2018). Beta-blockers for heart failure with reduced, mid-range, and preserved ejection fraction: an individual patient-level analysis of double-blind randomized trials. Eur Heart J.

[58] Hjalmarson Å., Goldstein S., Fagerberg B. (2000). Effects of controlled-release metoprolol on total mortality, hospitalizations, and well-being in patients with heart failure: the Metoprolol CR/XL Randomized Intervention Trial in Congestive Heart Failure (MERIT-HF). JAMA.

[59] Willenheimer R., Van Veldhuisen D.J., Silke B. (2005). Effect on survival and hospitalization of initiating treatment for chronic heart failure with bisoprolol followed by enalapril, as compared with the opposite sequence: results of the randomized Cardiac Insufficiency Bisoprolol Study (CIBIS) III. Circulation.

[60] Halliday B.P., Wassall R., Lota A.S. (2019). Withdrawal of pharmacological treatment for heart failure in patients with recovered dilated cardiomyopathy (TRED-HF): an open-label, pilot, randomised trial. Lancet.

[61] Waagstein F., Caidahl K., Wallentin I., Bergh C.H., Hjalmarson A. (1989). Long-term beta-blockade in dilated cardiomyopathy. Effects of short- and long-term metoprolol treatment followed by withdrawal and readministration of metoprolol. Circulation.

[62] Marti C.N., Fonarow G.C., Anker S.D. (2019). Medication dosing for heart failure with reduced ejection fraction - opportunities and challenges. Eur J Heart Fail.

[63] Kotecha D., Holmes J., Krum H. (2014). Efficacy of β blockers in patients with heart failure plus atrial fibrillation: an individual-patient data meta-analysis. Lancet.

[64] Flather M.D., Shibata M.C., Coats A.J.S. (2005). Randomized trial to determine the effect of nebivolol on mortality and cardiovascular hospital admission in elderly patients with heart failure (SENIORS). Eur Heart J.

[65] Pitt B., Remme W., Zannad F. (2003). Eplerenone, a selective aldosterone blocker, in patients with left ventricular dysfunction after myocardial infarction. N Engl J Med.

[66] Arnold W.P., Mittal C.K., Katsuki S., Murad F. (1977). Nitric oxide activates guanylate cyclase and increases guanosine 3′:5′-cyclic monophosphate levels in various tissue preparations. Proc Natl Acad Sci USA.

[67] Hardman J.G., Sutherland E.W. (1969). Guanyl cyclase, an enzyme catalyzing the formation of guanosine 3’,5’-monophosphate from guanosine triphosphate. J Biol Chem.

[68] Hardman J.G., Davis J.W., Sutherland E.W. (1969). Effects of some hormonal and other factors on the excretion of guanosine 3’,5’-monophosphate and adenosine 3’,5’-monophosphate in rat urine. J Biol Chem.

[69] McNamara D.B., Kadowitz P.J., Hyman A.L., Ignarro L.J. (1980). Adenosine 3’,5’-monophosphate formation by preparations of rat liver soluble guanylate cyclase activated with nitric oxide, nitrosyl ferroheme, S-nitrosothiols, and other nitroso compounds. Can J Physiol Pharmacol.

[70] Moncada S., Palmer R.M., Higgs E.A. (1988). The discovery of nitric oxide as the endogenous nitrovasodilator. Hypertension.

[71] Moncada S., Higgs E.A. (2006). Nitric oxide and the vascular endothelium. Handb Exp Pharmacol.

[72] Ambrosy A.P., Butler J., Ahmed A. (2014). The use of digoxin in patients with worsening chronic heart failure: reconsidering an old drug to reduce hospital admissions. J Am Coll Cardiol.

[73] Ahmed A., Rich M.W., Love T.E. (2006). Digoxin and reduction in mortality and hospitalization in heart failure: a comprehensive post hoc analysis of the DIG trial. Eur Heart J.

[74] Ahmed A., Pitt B., Rahimtoola S.H. (2008). Effects of digoxin at low serum concentrations on mortality and hospitalization in heart failure: a propensity-matched study of the DIG trial. Int J Cardiol.

[75] Aguirre Dávila L., Weber K., Bavendiek U. (2019). Digoxin-mortality: randomized vs. observational comparison in the DIG trial. Eur Heart J.

[76] Digitalis Investigation Group (1997). The effect of digoxin on mortality and morbidity in patients with heart failure. N Engl J Med.

[77] Adams K.F., Butler J., Patterson J.H. (2016). Dose response characterization of the association of serum digoxin concentration with mortality outcomes in the Digitalis Investigation Group trial. Eur J Heart Fail.

[78] Lopes R.D., Rordorf R., De Ferrari G.M. (2018). Digoxin and mortality in patients with atrial fibrillation. J Am Coll Cardiol.

[79] Malik A., Masson R., Singh S. (2019). Digoxin discontinuation and outcomes in patients with heart failure with reduced ejection fraction. J Am Coll Cardiol.

[80] Ather S., Chan W., Bozkurt B. (2012). Impact of noncardiac comorbidities on morbidity and mortality in a predominantly male population with heart failure and preserved versus reduced ejection fraction. J Am Coll Cardiol.

[81] Mentz R.J., Kelly J.P., Von Lueder T.G. (2014). Noncardiac comorbidities in heart failure with reduced versus preserved ejection fraction. J Am Coll Cardiol.

[82] Paulus W.J., Tschöpe C. (2013). A novel paradigm for heart failure with preserved ejection fraction: comorbidities drive myocardial dysfunction and remodeling through coronary microvascular endothelial inflammation. J Am Coll Cardiol.

[83] Pfeffer M.A., Claggett B., Assmann S.F. (2015). Regional variation in patients and outcomes in the treatment of preserved cardiac function heart failure with an aldosterone antagonist (TOPCAT) trial. Circulation.

[84] Bozkurt B., Savarese G., Adamsson Eryd S. (2023). Mortality, outcomes, costs, and use of medicines following a first heart failure hospitalization. J Am Coll Cardiol HF.

[85] Greene S.J., Butler J., Albert N.M. (2018). Medical therapy for heart failure with reduced ejection fraction: the CHAMP-HF registry. J Am Coll Cardiol.

[86] Deschaseaux C., McSharry M., Hudson E., Agrawal R., Turner S.J. (2016). Treatment initiation patterns, modifications, and medication adherence among newly diagnosed heart failure patients: a retrospective claims database analysis. J Manag Care Spec Pharm.

[87] Wirtz H.S., Sheer R., Honarpour N. (2020). Real-world analysis of guideline-based therapy after hospitalization for heart failure. J Am Heart Assoc.

[88] Savarese G., Kishi T., Vardeny O. (2023). Heart failure drug treatment-inertia, titration, and discontinuation: a multinational observational study (EVOLUTION HF). J Am Coll Cardiol HF.

[89] Mebazaa A., Davison B., Chioncel O. (2022). Safety, tolerability and efficacy of up-titration of guideline-directed medical therapies for acute heart failure (STRONG-HF): a multinational, open-label, randomised, trial. Lancet.

[90] Biegus J., Voors A.A., Collins S.P. (2023). Impact of empagliflozin on decongestion in acute heart failure: the EMPULSE trial. Eur Heart J.

[91] Rao V.N., Murray E., Butler J. (2021). In-hospital initiation of sodium-glucose cotransporter-2 inhibitors for heart failure with reduced ejection fraction. J Am Coll Cardiol.

[92] Peri-Okonny P.A., Mi X., Khariton Y. (2019). Target doses of heart failure medical therapy and blood pressure: insights from the CHAMP-HF registry. J Am Coll Cardiol HF.

[93] Greene S.J., Fonarow G.C., DeVore A.D. (2019). Titration of medical therapy for heart failure with reduced ejection fraction. J Am Coll Cardiol.

[94] Freedland K.E., Carney R.M., Rich M.W., Steinmeyer B.C., Skala J.A., Dávila-Román V.G. (2016). Depression and multiple rehospitalizations in patients with heart failure. Clin Cardiol.

[95] Gallagher R., Luttik M.L., Jaarsma T. (2011). Social support and self-care in heart failure. J Cardiovasc Nurs.

[96] Ahmed M.B., Patel K., Fonarow G.C. (2016). Higher risk for incident heart failure and cardiovascular mortality among community-dwelling octogenarians without pneumococcal vaccination. ESC Heart Fail.

[97] Sandhu A.T., Heidenreich P.A. (2021). The affordability of guideline-directed medical therapy: cost sharing is a critical barrier to therapy adoption. Circulation.

[98] Alkhatib D., Isa S., Pour-Ghaz I. (2023). Novel device therapies for heart failure. J Cardiovasc Dev Dis.

[99] Martens P., Burkhoff D., Cowger J.A., Jorde U.P., Kapur N.K., Tang W.H.W. (2023). Emerging individualized approaches in the management of acute cardiorenal syndrome with renal assist devices. J Am Coll Cardiol HF.

[100] Salah H.M., Levin A.P., Fudim M. (2022). Device therapy for heart failure with preserved ejection fraction. Cardiol Clin.

[101] Cerrud-Rodriguez R.C., Burkhoff D., Latib A., Granada J.F. (2022). A glimpse into the future of transcatheter interventional heart failure therapies. J Am Coll Cardiol Basic Trans Science.

[102] Mastoris I., Spall H.G.C.V., Sheldon S.H. (2022). Emerging implantable-device technology for patients at the intersection of electrophysiology and heart failure interdisciplinary care. J Card Fail.

[103] Givertz M.M., Stevenson L.W., Costanzo M.R. (2017). Pulmonary artery pressure-guided management of patients with heart failure and reduced ejection fraction. J Am Coll Cardiol.

[104] Radhoe S.P., Veenis J.F., Brugts J.J. (2021). Invasive devices and sensors for remote care of heart failure patients. Sensors (Basel).

[105] Kennel P.J., Rosenblum H., Axsom K.M. (2022). Remote cardiac monitoring in patients with heart failure: a review. JAMA Cardiol.

[106] Curtis A.B. (2013). Biventricular pacing for atrioventricular block and systolic dysfunction. N Engl J Med.

[107] Doshi R.N., Daoud E.G., Fellows C. (2005). Left ventricular-based cardiac stimulation post AV nodal ablation evaluation (the PAVE study). J Cardiovasc Electrophysiol.

[108] Pugh T.J., Kelly M.A., Gowrisankar S. (2014). The landscape of genetic variation in dilated cardiomyopathy as surveyed by clinical DNA sequencing. Genet Med.

[109] Gigli M., Merlo M., Graw S.L. (2019). Genetic risk of arrhythmic phenotypes in patients with dilated cardiomyopathy. J Am Coll Cardiol.

[110] Mohri S., He K.L., Dickstein M. (2002). Cardiac contractility modulation by electric currents applied during the refractory period. Am J Physiol Heart Circ Physiol.

[111] Mohri S., Shimizu J., Mika Y. (2003). Electric currents applied during refractory period enhance contractility and systolic calcium in the ferret heart. Am J Physiol Heart Circ Physiol.

[112] Borggrefe M., Mann D.L. (2018). Cardiac contractility modulation in 2018. Circulation.

[113] Sharif Z.I., Galand V., Hucker W.J., Singh J.P. (2021). Evolving cardiac electrical therapies for advanced heart failure patients. Circ Arrhythm Electrophysiol.

[114] Yano M., Ikeda Y., Matsuzaki M. (2005). Altered intracellular Ca2+ handling in heart failure. J Clin Invest.

[115] Giallauria F., Cuomo G., Parlato A., Raval N.Y., Kuschyk J., Stewart Coats A.J. (2020). A comprehensive individual patient data meta-analysis of the effects of cardiac contractility modulation on functional capacity and heart failure-related quality of life. ESC Heart Fail.

[116] Kaufmann H., Norcliffe-Kaufmann L., Palma J.A. (2020). Baroreflex dysfunction. N Engl J Med.

[117] Hoppe U.C., Brandt M.C., Wachter R. (2012). Minimally invasive system for baroreflex activation therapy chronically lowers blood pressure with pacemaker-like safety profile: results from the Barostim neo trial. J Am Soc Hypertens.

[118] Georgakopoulos D., Little W.C., Abraham W.T., Weaver F.A., Zile M.R. (2011). Chronic baroreflex activation: a potential therapeutic approach to heart failure with preserved ejection fraction. J Card Fail.

[119] Paolisso P., Dagan A., Gallinoro E. (2023). Aortic thoracic neuromodulation in heart failure with preserved ejection fraction. ESC Heart Fail.

[120] Bartunek J. Aortic arch simulation (Enopace). March 21, 2023. https://www.tctmd.com/slide/aortic-arch-simulation-enopace.

[121] Spiering W., Williams B., Van Der Heyden J. (2017). Endovascular baroreflex amplification for resistant hypertension: a safety and proof-of-principle clinical study. Lancet.

[122] Sievert H. Mechanical stimulation of the Carotid Sinus: MobiusHD. March 21, 2023. https://www.tctmd.com/slide/mechanical-stimulation-carotid-sinus-mobiushd.

[123] Piayda K., Sievert K., Sievert H. (2022). Endovascular baroreflex amplification with the MobiusHD device in patients with heart failure and reduced ejection fraction: interim analysis of the first-in-human results. Struct Heart.

[124] Obadia J.F., Messika-Zeitoun D., Leurent G. (2018). Percutaneous repair or medical treatment for secondary mitral regurgitation. N Engl J Med.

[125] Pibarot P., Delgado V., Bax J.J. (2019). MITRA-FR vs. COAPT: lessons from two trials with diametrically opposed results. Eur Heart J Cardiovasc Imaging.

[126] Topilsky Y., Maltais S., Medina Inojosa J. (2019). Burden of tricuspid regurgitation in patients diagnosed in the community setting. J Am Coll Cardiol Img.

[127] Nanayakkara S., Kaye D.M. (2023). Device therapy with interatrial shunt devices for heart failure with preserved ejection fraction. Heart Fail Rev.

[128] Riccardi M., Tomasoni D., Vizzardi E., Metra M., Adamo M. (2023). Device-based percutaneous treatments to decompress the left atrium in heart failure with preserved ejection fraction. Heart Fail Rev.

[129] Hasenfuß G., Hayward C., Burkhoff D. (2016). A transcatheter intracardiac shunt device for heart failure with preserved ejection fraction (REDUCE LAP-HF): a multicentre, open-label, single-arm, phase 1 trial. Lancet.

[130] Litwin S.E., Komtebedde J., Hu M. (2023). Exercise-induced left atrial hypertension in heart failure with preserved ejection fraction. J Am Coll Cardiol HF.

[131] Del Trigo M., Bergeron S., Bernier M. (2016). Unidirectional left-to-right interatrial shunting for treatment of patients with heart failure with reduced ejection fraction: a safety and proof-of-principle cohort study. Lancet.

[132] Udelson J.E., Barker C.M., Wilkins G. (2023). No-implant interatrial shunt for HFpEF: 6-month outcomes from multicenter pilot feasibility studies. J Am Coll Cardiol HF.

[133] Salah H.M., Butler J., Fudim M. (2023). Rapid sequence initiation of device therapy in heart failure. JACC: Adv.

[134] Leyva F., Zegard A., Patel P. (2023). Timing of cardiac resynchronization therapy implantation. Europace.

