# Supplementary material for: Medical Management and Device-Based Therapies in Chronic Heart Failure
Source: J Soc Cardiovasc Angiogr Interv. 2023 Dec 4;2(6Part B):101206. doi: 10.1016/j.jscai.2023.101206 (PMC11308856; doi:10.1016/j.jscai.2023.101206)
Supplement: Supplementary Material [file mmc1.docx]

Figure 2 Citations:

- <https://www.cardiovascular.abbott/us/en/hcp/products/heart-failure/pulmonary-pressure-monitors/cardiomems/procedure-overview.html> (CardioMEMs)
- <https://mitraclip.com/physician/mitraclip-procedure/mitraclip-tmvr-mitral-regurgitation-treatment> (Mitraclip)
- <https://www.structuralheart.abbott/int/products/transcatheter-tricuspid-valve-repair/triclip-tmvr-teer> (Triclip)
- <https://www.ahajournals.org/doi/10.1161/CIRCULATIONAHA.105.566885> (CRT)
- <https://impulse-dynamics.com/providers/> (CCM)
- <https://www.tctmd.com/news/fda-approves-baroreflex-activation-therapy-device-advanced-heart-failure> (BAT)
- <https://corviamedical.com/corvia-atrial-shunt-system/> (Interatrial shunt)
